# A Nonlinear Causality Estimator Based on Non-Parametric Multiplicative Regression

**DOI:** 10.3389/fninf.2016.00019

**Published:** 2016-06-14

**Authors:** Nicoletta Nicolaou, Timothy G. Constandinou

**Affiliations:** Department of Electrical and Electronic Engineering, Imperial College LondonLondon, UK

**Keywords:** nonparametric multiplicative regression, nonlinear causality, nonparametric causality, multivariate causality, conditional causality

## Abstract

Causal prediction has become a popular tool for neuroscience applications, as it allows the study of relationships between different brain areas during rest, cognitive tasks or brain disorders. We propose a nonparametric approach for the estimation of nonlinear causal prediction for multivariate time series. In the proposed estimator, *C*_*NPMR*_, Autoregressive modeling is replaced by Nonparametric Multiplicative Regression (NPMR). NPMR quantifies interactions between a response variable (effect) and a set of predictor variables (cause); here, we modified NPMR for model prediction. We also demonstrate how a particular measure, the sensitivity Q, could be used to reveal the structure of the underlying causal relationships. We apply *C*_*NPMR*_ on artificial data with known ground truth (5 datasets), as well as physiological data (2 datasets). *C*_*NPMR*_ correctly identifies both linear and nonlinear causal connections that are present in the artificial data, as well as physiologically relevant connectivity in the real data, and does not seem to be affected by filtering. The Sensitivity measure also provides useful information about the latent connectivity.The proposed estimator addresses many of the limitations of linear Granger causality and other nonlinear causality estimators. *C*_*NPMR*_ is compared with pairwise and conditional Granger causality (linear) and Kernel-Granger causality (nonlinear). The proposed estimator can be applied to pairwise or multivariate estimations without any modifications to the main method. Its nonpametric nature, its ability to capture nonlinear relationships and its robustness to filtering make it appealing for a number of applications.

## 1. Introduction

Causality was introduced by Wiener (1956) and mathematically formulated by Granger to study cause and effect between variables for econometric applications (Granger, 1969). Formally, causality quantifies interactions between variables and identifies cause-effect relationships through modeling, prediction and assessment of the goodness-of-fit when past information from one variable (cause) are incorporated into the prediction of another variable (effect). Granger causality is quantified from the goodness-of-fit of Autoregressive models fitted onto the effect on its own (univariate model), and fitted onto the effect and the cause together (bivariate model). Causality has become a popular tool in a number of diverse fields (e.g., see Freeman, 1983; Oh and Lee, 2004; Sugihara et al., 2012; Lu et al., 2014). Applications in neuroscience, which is our particular field of interest, have been gaining ground over the last decade (e.g., Kaminski et al., 2001; Hesse et al., 2003; Chen et al., 2006; Nicolaou et al., 2012).

Since the introduction of Granger causality there have also been a number of modifications and extensions to the basic formulation (for a review see Ding et al., 2006; Guo et al., 2010). However, the majority of extensions remain faithful to the traditional approach of AR-based modeling and are only applicable to linear causality. In the current study we are particularly interested in nonlinear extensions.

Faes et al. (2008a) proposed a causality estimator based on nonlinear exogenous autoregressive (NARX) modeling. This nonlinear estimator uses Optimal Parameter Search for NARX parameter estimation, which is less dependent on the initial choice of model order, and a directionality index to quantify the strength of causal interactions (based on the mean squared prediction error of the fitted models). The estimator was shown to capture linear and nonlinear causality on artificial and physiological data (cardiovascular interactions). However, the method remains parametric and is only applicable to bivariate interactions. An additional drawback of this estimator, identified by the authors themselves, is that the estimator is only appropriate for nonlinearities up to 3rd order only; otherwise, the number of model parameters that need to be searched becomes computationally prohibiting. Chen et al. (2004) propose another nonlinear extension to Granger causality, extended Granger causality (a similar technique was also proposed in Freiwald et al., 1999). This method is based on fitting locally linear models (AR) to randomly selected neighborhoods of the embedded time-series and estimating the neighborhood Granger causality. Extended Granger causality is then estimated as the average of this local GC. It is applicable for bivariate, as well as conditional estimations. The method still uses AR modeling and the optimal neighborhood size is an additional consideration. The trade-off is that the neighborhood should be large enough to obtain a representative estimate, but small enough to ensure valid linearization (Chen et al., 2004).

Some approaches move away from traditional AR-based estimators. Ancona et al. (2004) propose a bivariate nonlinear causality estimator, which replaces AR with a Radial Basis Function (RBF)-like approach to prediction. The estimator performance was demonstrated on artificial data (unidirectionally coupled chaotic maps) and physiological data (heart and breath rate during apneic sleep, and rat epileptic EEG). The method remains parametric and has the following additional limitations: (i) the centers (i.e., mean values) that are used in the RBF-like models are found via clustering, adding yet another parametric constraint to the process; and (ii) the estimator is developed for bivariate causality and there is no discussion by the authors whether an extension to conditional/multivariate estimation is feasible. Marinazzo et al. (2008b) propose the Kernel-Granger causality estimator, initially for bivariate and later extended to multivariate estimations (Marinazzo et al., 2008a). Kernel-Granger causality performs linear Granger causality (vector autoregression) in the feature space of the kernel function; thus, the nonlinearity of the regression model is controlled by the choice of the kernel. Granger causality analysis (linear regression) is then performed in the space constructed by the inner product of the inputs and the (nonlinear) kernel function. This space is spanned by the eigenvectors of the kernel and regression is equivalent to the correlation of the input data and these eigenvectors. The causality index is estimated as the mean of the statistically significant correlations. Kernel-GC does not suffer from the problem of overfitting and is applicable to multivariate data. Even though in theory any appropriate kernel function can be used, the geometrical approach must be altered first (Marinazzo et al., 2008b). In addition, there are some types of nonlinearities that cause Kernel-GC performance to degrade, e.g., if the data contain nonlinearities caused by limits on the upper and lower amplitude (Marinazzo et al., 2008a) or increasing amount of noise (Marinazzo et al., 2011).

Yet another approach is that by Schiff et al. (1996) based on nonlinear mutual prediction. This is not a measure of causality *per se*, but more a measure of generalized synchrony that can be used to infer the direction of information flow. Schiff et al. use nonlinear mutual prediction to characterize the evolution of a system trajectory over a prediction horizon. This is done by tracking how the *n* nearest neighbors of a particular time point evolve in this prediction horizon and comparing this projection with the actual evolution of the time point. This can be extended to mutual prediction of 2 time series by looking at how their mutual neighbors evolve over time and can be used to infer the directionality of prediction between the two systems. The proposed measure is bivariate and has some limitations when the coupling is bidirectional. A comparison of different existing conceptual approaches to mutual prediction of bivariate time series is presented in Faes et al. (2008b). The three approaches compared were based on the k-nearest neighbor local linear approximation (Farmer and Sidorowich, 1987) (for another study comparing k-nearest neighbor-based causality estimators see Krakovská et al., 2015). From the comparison of Faes and colleagues, it is evident that the choice of a particular estimation strategy is guided by knowledge of the ground truth. If one does not know the ground truth, then it is difficult to choose a particular strategy as the results obtained are inconsistent. For example, it is difficult to infer with certainty whether a significant cross-prediction value is uni- or bi-directional, or to interpret the results when mixed prediction is used, unless the ground truth is known (Faes et al., 2008b).

We would briefly also like to mention another class of methods based on graphical causal inference, following the work by Pearl and colleagues who combine causal models with causal graphs Pearl (2009). In Pearl's causality framework a causal model is composed of a set of variables, both endogenous and exogenous to the model (exogenous variables are considered as noise terms). The model can be expressed as a function (linear or nonlinear) of a subset of all variables and a set of constant parameters, and can be represented graphically with endogenous variables corresponding to graph nodes connected by directed acyclic edges representing causal connections. This class of methods is a non-parametric approach for inferring causal connections from empirical observations, even when latent variables are present. A downside of this approach is that it involves a probabilistic approach based on Markov factorization, i.e., temporal structure and causal relationships originating from samples further in the past are ignored. These models have been extended to time-series causal modeling (see Arnold et al., 2007 for a comparison and Chicharro and Panzeri, 2014 for a specific application to brain signal analysis). The copula approach is a well-known nonlinear extension to graphical causal inference (Bahadori and Liu, 2013); however, this is a semi-parametric method.

An additional consideration is the general problem of overfitting, which the majority of current approaches in nonlinear causality estimation suffer from. As Palu$\overset{}{s}$ and Vejmeka point out, the particular problem leads to the detection of false causalities (Palus and Vejmeka, 2007). Only some of the techniques described above do not suffer from overfitting, e.g., Kernel Granger causality.

We propose here an estimator that follows the traditional Granger causality methodology, but the univariate/multivariate linear/nonlinear AR models are replaced with Non-Parametric Multiplicative Regression (NPMR). The proposed estimator, *C*_*NPMR*_, addresses the following limitations of existing causality estimators (summarized in Table l): (1) it is nonparametric, therefore, estimation is guided by the data itself as opposed to an underlying parametric model of specific form; (2) it can detect both linear and nonlinear causality; (3) the multiplicative relationship between predictors means that the same method can be used without any modification for pairwise or conditional/multivariate causality estimation; (4) there is no restriction to the order of nonlinearity that can be estimated; and (5) it allows for immediate inclusion of new points in the model as these become available. These inherent features of NPMR can also be combined with the following procedures, in order to obtain a set of properties that are unique to the proposed estimator: (1) overfitting is addressed by using leave-one-out cross validation; (2) statistical significance can be readily assessed with surrogate data methods; and (3) the sensitivity measure *Q*, can be used to assess the relative importance of a particular predictor within a model. It may be possible to use this measure to infer the underlying structure of the causal relationships that is otherwise unknown and non-observable. The proposed method has some similarities to existing nonparametric methods that use smoothing functions, such as Radial Basis Functions and General Additive Models, as well as with Kernel-Granger causality. These similarities arise mostly when the same smoothing function or kernel function is employed; however, a main difference is the multiplicative rather than additive combination of weights in NPMR (more details can be found in the Discussion).

**Table 1:** **Comparison of causality methodologies**.

**Citation**	**Pairwise**	**Multivariate**	**Linear**	**Non-linear**	**Parametric**	**Nonparametric**	**Other**
Granger, 1969	✓	—	✓	—	✓	—	Problems with indirect connections
Faes et al., 2008a	✓	—	✓	✓	✓	—	Only for non-linearities < 3rd order
Chen et al., 2004	✓	✓	✓	✓	✓	—	Needs lots of data
Ancona et al., 2004	✓	—	✓	✓	✓	—	
Schiff et al., 1996	✓	—	✓	✓	✓	—	Issues with bidirectional coupling
Proposed	✓	✓	✓	✓	—	✓	No overfitting

The remainder of this paper is structured as follows. Section 2 introduces Non-Parametric Multiplicative Regression and how this can be adapted for causality estimation. Section 3 presents the results of testing the performance of the proposed causality estimator on artificial data (with linear and nonlinear causal effects) and physiological data (cardiovascular effects during sleep and EEG activity during anesthesia). Additional considerations are given in Section 4.

## 2. Materials and methods

For two time series, *X* and *Y*, Granger causality is mathematically defined as the ratio of the univariate and bivariate Autoregressive (AR) model fitting errors:

(1) $$\begin{array}{l} GC\left(Y\to X\right)=log\left(\frac{\sigma_{X∕X}^{2}}{\sigma_{X∕\left(X,Y\right)}^{2}}\right) \end{array}$$

where $\sigma_{X∕X}^{2}$ and $\sigma_{X∕\left(X,Y\right)}^{2}$ are the variances of the prediction errors of the univariate and bivariate AR models respectively. The parametric nature of Granger causality (GC) is also one of its main limitations. Its dependence on the use of AR modeling, whether this is linear/nonlinear or bivariate/multivariate, imposes a model of a particular form onto the data. Even though AR models are quite versatile they do not always capture the underlying data structure accurately. The same holds for any other modifications to GC that still rely on fitting a parametric model to the data.

In this work we have replaced the modeling part of GC with a non-parametric, nonlinear regression-based method. Non-Parametric Multiplicative Regression (NPMR) originates from the field of ecology and was first introduced for habitat modeling in McCune (2006, 2011). A habitat model describes how variations in species performance relate to different predictors, such as environmental variables and site characteristics. In this context, NPMR is a method that seeks and characterizes relationships (linear and nonlinear) between species performance and specific predictors without explicitly estimating any coefficients in a fixed mathematical form. Instead, the method finds an optimized fit of the data on a local model, e.g., local mean estimation or local linear regression. The contribution of each predictor to this local function is weighted via a kernel function, e.g., Gaussian kernel. The outcome of NPMR application is to identify which predictors have the largest and most important effect on species performance. This is down to the multiplicative nature of NPMR: the weights for individual predictors are combined multiplicatively rather than additively and this allows for interactions between the individual predictors (McCune, 2006). The combined weight at a given point can become zero if any of the predictor variables fails with respect to similarity with that particular point.

In the proposed estimator of causality, *C*_*NPMR*_, the univariate and bivariate/multivariate AR models are replaced by the equivalent univariate and bivariate/multivariate NPMR model. Causality is then estimated following the formal mathematical definition of causality (Equation 1).

### 2.1. Non-parametric multiplicative regression

The idea behind Non-Parametric Multiplicative Regression (NPMR) can be extended to applications outside of habitat modeling. In the context of time-series modeling, NPMR can be used to identify a set of variables that are important in modeling a particular time-series. However, NPMR can be modified such that information about the past of a set of variables is also included in the modeling of a time-series. This is similar to how an AR model is based on past information. The quality of the model fit can be assessed through the variance of the error of the fit.

Firstly, we will describe the basic idea of NPMR. Denote a variable to be modeled (response variable), *Y* = {*y*_1_, *y*_2_, …, *y*_*T*_} (with *T* samples) and a matrix of *m* predictors,

$$X=\left(\begin{array}{cccc} x_{1,1} & x_{1,2} & \cdots & x_{1,m}\\ x_{2,1} & x_{2,2} & \cdots & x_{2,m}\\ \vdots & \vdots & \ddots & \vdots \\ x_{T,1} & x_{T,2} & \cdots & x_{T,m} \end{array}\right)$$

NPMR builds a global response surface of *Y* from its relationship with the *m* predictors **X**. This is achieved by estimating the value of *y*_*t*_(*t* = 1, …*T*) from information from the local neighborhood of the corresponding point in the predictor space, **X_t_** = [*x*_*t*, 1_, …, *x*_*t, m*_], using a multiplicative kernel smoother. The influence of each of the *m* predictors on this estimate is defined by the tolerance of the kernel smoother (*s*_*j*_; *j* = 1, …, *m*), i.e., neighborhood size. The ability to define a different tolerance for each predictor is unique to NPMR. In this example (and throughout this study) the local neighborhood is defined as the weighted mean and the weights are estimated from the simple and intuitive Gaussian kernel smoother. Estimation of *y*_*t*_ is, thus, obtained as:

(2) $$\begin{array}{l} {ŷ}_{t}=\frac{\overset{T}{\underset{i=1,i\neq t}{\sum }}y_{i}\left(\overset{m}{\underset{j=1}{\prod }}w_{ij}\right)}{\overset{T}{\underset{i=1,i\neq t}{\sum }}\left(\overset{m}{\underset{j=1}{\prod }}w_{ij}\right)} \end{array}$$

In Equation (2), we see that the point of interest, *t*, is omitted from the estimation. This leave-one-out cross validation avoids overfitting. The weights, *w*_*i, j*_, are the distances of each of the *m* predictors to the target point **X**_*t*_, estimated with a Gaussian kernel weighting function with tolerance (standard deviation) σ_*j*_ and centered at the point of interest, *t*:

(3) $$\begin{array}{l} w_{i,j}=e^{-0.5{\left[\left(x_{i,j}-x_{t,j}\right)∕\sigma_{j}\right]}^{2}} \end{array}$$

The Gaussian kernel tolerance (standard deviation, σ) defines how rapidly the weights diminish with distance from the target point. Following McCune (2011), the tolerance can be optimized via an iterative search; for the Gaussian kernel it could also be estimated from the data itself such that the range of the observed values for a specific predictor fall within six standard deviations. Even though there are no guidelines on the use of a particular Kernel function, the Gaussian kernel provides a simple and flexible way of expressing the weight of a point as a function of its distance from the target. As discussed in McCune (2011), the Gaussian kernel considers all available points in the target prediction while allowing the weights to decrease smoothly for observations further from the target, and the speed of this decrease can be easily controlled with the kernel tolerance. If, however, one wants to take into account only points that are within a specified neighborhood of the target point, then a rectangular kernel would be preferable.

A detailed numerical example of NPMR is provided in Supplementary Material Appendix 1; see also McCune (2011).

### 2.2. NPMR-based causality estimation

We extend the basic idea of NPMR for causality estimation. The predictor space is modified to include past information as additional predictors. This could be past information of the variable to be modeled (univariate prediction) or information from both the variable to be modeled and chosen predictor variables (bivariate/multivariate prediction). Including past information is achieved through Takens' time-delay embedding (Takens, 1981). For a point, *z*_*t*_, its time-delay embedded equivalent, ${\tilde{z}}_{t}$, with delay τ and embedding dimension *d* is:

(4) $$\begin{array}{l} {\tilde{z}}_{t}=\left\{z_{t},z_{t-\tau },z_{t-2\tau },\ldots ,z_{t-\left(d-1\right)\tau }\right\} \end{array}$$

This well-known theorem allows reconstruction of the system dynamics that are not readily available from the observed time series alone. By time-delay embedding the response and/or predictor variables, we effectively include past information in the estimates of ŷ_*t*_. NPMR can then be applied to *Y* using its time-delayed equivalent $\tilde{\text{Y}}$ as predictors (univariate prediction) or combining its time-delayed equivalent $\tilde{\text{Y}}$ with the time-delayed predictor variables, $\tilde{\text{X}}$ (bivariate/multivariate prediction). By choosing which predictors are included in the modeling one can then assess the significance (not in strict statistical sense) of each predictor through the variance of the modeling error: smaller variance indicates better goodness of fit. Supplementary Material Appendix 1 provides a numerical example of how NPMR can be used for time-series prediction.

Putting this into a causality framework one can assess the existence of causal relationships using the standard Granger causality definition, with the error variances replaced by those obtained from NPMR modeling. Thus, for a bivariate estimate the NPMR-based causality, *C*_*NPMR*_(*X*_*i*_ → *Y*), can be estimated as:

(5) $$\begin{array}{l} C_{NPMR}\left(X_{i}\to Y\right)=log\left(\frac{\sigma_{\left(Y,\tilde{\text{Y}}\right)}^{2}}{\sigma_{\left(Y,\left(\tilde{\text{Y}},X_{i}\right)\right)}^{2}}\right) \end{array}$$

where $\sigma_{\left(Y,Ỹ\right)}^{2}$ and $\sigma_{\left(Y,\left(Ỹ,{\tilde{X}}_{i}\right)\right)}^{2}$ are the error variances when past values of *Y* or past values of both *Y* and *X*_*i*_ are used in the prediction of *Y* respectively. In the traditional Granger causal sense this would correspond to the residuals from univariate and bivariate autoregressive models and Equation (5) is equivalent to pairwise causality estimation.

The methodology can readily be extended to conditional causality estimation. The formal conditional definition of *C*_*NPMR*_ follows that of standard conditional Granger causality:

(6) $$C_{NPMR}(X_{i}\to Y/Z)=log\left(\frac{\sigma_{\left(Y,(\tilde{Y},Z)\right)}^{2}}{\sigma_{\left(Y,((\tilde{Y},{\tilde{X}}_{i},Z)\right)}^{2}}\right)$$

where **Z** is a matrix of all predictors we would like to include in the conditional model, apart from *X*_*i*_. Causalities in the opposite direction are estimated by switching the response and predictor variables. The above definitions of *C*_*NPMR*_ are not bounded. This means that it is also possible to obtain negative *C*_*NPMR*_ values. Negative values imply that the addition of past information from other time-series results in worse model fit and, therefore, there is no significant causal relationship between the time-series.

### 2.3. Sensitivity and model fit

The relative importance of particular predictors within an NPMR model can be assessed with sensitivity analysis. Sensitivity analysis involves the addition/subtraction of small noise to individual predictors and measuring the resulting change in the estimated response. The change in the response, ŷ^+^ and ŷ^−^, when the predictor is nudged up or down respectively can be used to estimate the sensitivity of the response, *Y*, to the jth predictor, *Q*(*Y*∕*X*_*j*_):

(7) $$Q(Y/X_{j})=\frac{\sum_{i=1}^{T}\left|{\hat{y}}_{i}^{+}-{\hat{y}}_{i}|+|{\hat{y}}_{i}^{-}-{\hat{y}}_{i}\right|}{2T|y_{max}-y_{min}|\Delta }$$

where *y*_*max*_ (*y*_*min*_) are the maximum (minimum) values of the response variable *Y*, ŷ_*i*_ is the target response estimated from Equation (2), and Δ is an arbitrarily small proportion by which the predictor will be nudged (0.05, i.e., a 5% change, is a commonly used value and has been used throughout this study). The notation *Q*(*Y*∕*X*_*j*_) and *Q*(*X*_*j*_ → *Y*) will also be used interchangeably. A value of *Q*(.) = 0 means that nudging a predictor has no detectable effect on the response; *Q*(.) = 1 means that, on average, nudging the predictor results in an equal change in the response magnitude; larger values of *Q*(.) correspond to higher sensitivity of the response to the particular predictor. The sensitivity measure could potentially allow us to identify underlying relationships in the analyzed time series that are otherwise unavailable and inaccessible with other causality estimators.

Supplementary Material Appendix 2 provides a numerical example of estimating the sensitivity.

In addition to the sensitivity, the relative importance of a predictor is also reflected in the overall model fit. NPMR models do not have explicit parameters and, thus, formal criteria that depend on the number of parameters in a model, such as the Akaike Information Criterion and the Bayesian Information Criterion, are not applicable. McCune (2011) recommends an alternative procedure for assessing the model fit. For quantitative variables, model quality can be evaluated via the “cross *R*^2^,”

(8) $$\begin{array}{l} xR^{2}=1-\frac{RSS}{TSS}=1-\frac{\overset{n}{\underset{i=1}{\sum }}{\left(y_{i}-{ŷ}_{i}\right)}^{2}}{\overset{n}{\underset{i=1}{\sum }}{\left(y_{i}-{ȳ}_{i}\right)}^{2}} \end{array}$$

where *y*_*i*_ is the response variable, ŷ_*i*_ is the estimated response variable and ȳ_*i*_ is the mean of the response variable. Equation 8 evaluates the model fit in terms of the residual sum-of-squares (RSS) and its relationship to the total sum of squares (TSS). These differ from traditional sum-of-squares measures as they are obtained from estimates via cross-validation (Equation 2). An *xR*^2^ value close to 1 denotes a good model fit, with quality of model fit dropping as *xR*^2^ approaches 0. For very weak models it is also possible to obtain negative *xR*^2^ values.

### 2.4. Estimator performance assessment

To investigate the behavior of the proposed estimator we use artificially generated signals from models where the ground truth is known. The models include linear and nonlinear causalities (datasets 1–5). We also apply the estimator on two physiological datasets: (i) cardiovascular interactions in sleep (dataset 6); the particular dataset has also been analyzed in related studies, the findings of which will form a basis for assessing the proposed estimator, and (ii) brain connectivity changes during wakefulness and anesthesia (dataset 7).

Statistical significance was assessed using surrogate data. The concept of surrogate methods is to break up any existing relationships in the data. There are various ways of achieving this and the method of time-shifted surrogates was used here, a surrogate data generation method that is appropriate for assessing nonlinear relationships (Quiroga et al., 2002). In order to generate a surrogate predictor variable the original predictor variable is shifted by *T* samples, where *T* is randomly chosen to be larger than approximately 1∕3 of the predictor variable length. The number of surrogate signals necessary for a particular significance level can be estimated via $S=\frac{1}{\alpha }-1$, where α is the desired statistical level. For example, for a 95% significance level (i.e., α = 0.05), 19 surrogate signals must be generated. The estimator is then applied to each pair of surrogate signals and the significance level is obtained as the maximum value of all *S* surrogate estimates. This acts as a threshold: if the real causality estimate exceeds this threshold, then this is considered as statistically significant. Regarding the sensitivity, only those values corresponding to significant *C*_*NPMR*_ are considered. Thus, there is no need to perform separate significance testing for the sensitivity. In addition, any negative *C*_*NPMR*_ values are rounded up to zero, since negative values are indicative of better univariate than bi/multi-variate model fit.

For all *C*_*NPMR*_ estimations we utilized a Gaussian kernel with tolerance σ_*j*_ = 1 for all predictors *j* = 1, …, *m*, time delay τ = 1 and embedding dimension *d* = 3 (in this study we did not use the *xR*^2^ measure to assess the model fit for different embedding dimensions; more details on how *xR*^2^ could be used, as well as on the choice of the tolerance and embedding parameters are given in the Discussion). For comparison purposes, we also estimated the (i) pairwise and conditional linear GC (conditional GC was estimated using the Granger causality estimation toolbox by Luo et al. (2013b), available online from http://www.dcs.warwick.ac.uk/~feng/causality.html); and the (ii) Kernel Granger causality (code available online from D. Marinazzo at http://users.ugent.be/~dmarinaz/KGC.html). For linear GC significance was assessed using the *F*-test (usual method of assessing significance of GC values). For K-GC significance is inherent in the methodology (only significant correlations are taken into account in the estimation of the K-GC value) and, therefore, no additional statistical method is used. Datasets were not normalized (z-transform) unless stated otherwise.

## 3. Results

### 3.1. Artificial data

#### 3.1.1. Dataset 1: unidirectional non-linear model

As a proof of principle, the estimator was initially applied on a simple model of two non-linearly interacting systems:

(9) $$\begin{array}{l} \begin{array}{c} x_{1}\left(t\right)=0.8x_{1}\left(t-1\right)+0.65x_{2}{\left(t-1\right)}^{2}+e_{1}\left(t\right)\\ x_{2}\left(t\right)=0.6x_{2}\left(t-1\right)+e_{2}\left(t\right) \end{array} \end{array}$$

where *e*_*i*_ (*i* = 1, 2) is normally distributed random noise. The model represents the unidirectional nonlinear causal effect *x*_2_ → *x*_1_. We generated 50 different realizations of 1000 samples from this model and estimated *C*_*NPMR*_(*x*_1_ ↔ *x*_2_) for each of the different realizations.

The mean causality (standard deviation) was only significant in the direction *x*_2_ → *x*_1_, with *C*_*NPMR*_(*x*_2_ → *x*_1_) = 0.357 (0.111), while *C*_*NPMR*_(*x*_1_ → *x*_2_) = −0.003 (0.013). For comparison purposes the corresponding Kernel-Granger causality values were: K-GC(*x*_1_ → *x*_2_) ~ 0 (0.002) and K-GC(*x*_2_ → *x*_1_) = 0.620 (0.026). Linear GC identified neglibible causality in the correct direction: GC(*x*_1_ → *x*_2_) = 0.003 (0.006) and GC(*x*_2_ → *x*_1_) = 0 (0).

We also introduce here how the sensitivity, *Q*, of the NPMR model, could provide information about the underlying structure of the interaction *x*_2_ → *x*_1_. From the sensitivity *Q*(*x*_1_ ∕ *x*_2_) we can deduce that the relationship between the two variables is mainly mediated by *x*_2_(*t* − 1):

1. *Q*(*x*_1_(*t*) ∕ *x*_2_(*t* − 1)) = 0.1095
2. *Q*(*x*_1_(*t*) ∕ *x*_2_(*t* − 2)) = 0.0429
3. *Q*(*x*_1_(*t*) ∕ *x*_2_(*t* − 3)) = 0.0289

#### 3.1.2. Dataset 2: multivariate model

**Figure 1:**
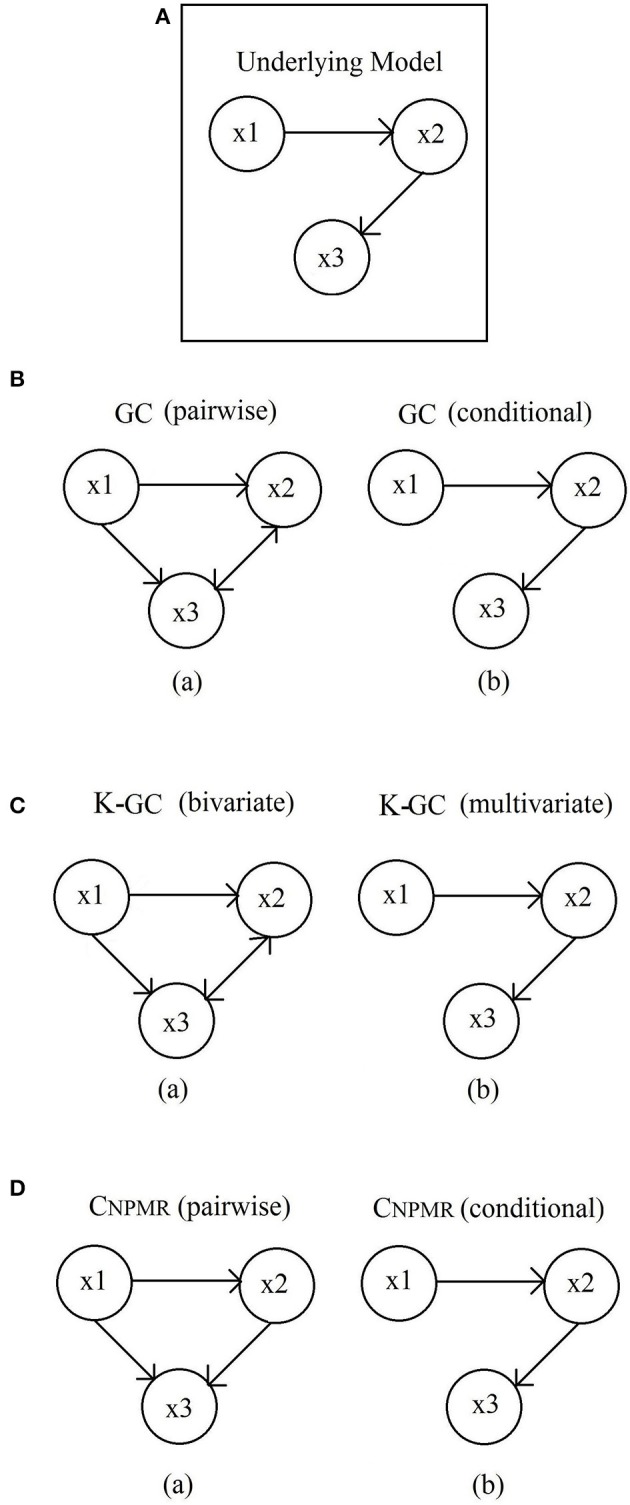
**Dataset 2: (A)** Underlying model. **(B)** Significant linear Granger causality. (a) Model estimated from pairwise *GC*; and (b) Model estimated from conditional *GC*. Pairwise *GC* identifies the indirect connection *x*_1_ → *x*_3_ as significant but also indicates the connection *x*_3_ → *x*_2_ as significant. **(C)** Kernel-Granger causality. (a) Bivariate and (b) Multivariate K-GC. For pairwise K-GC we observe similar behavior as with linear GC. **(D)** Significant *C*_*NPMR*_ directions identified from (a) pairwise and (b) conditional method. A significant connection from *x*_1_ → *x*_3_ is identified in the pairwise model. This indirect connection correctly vanishes for the conditional model.

The following multivariate model (Figure 1A) has both direct (*x*_1_ → *x*_2_, *x*_2_ → *x*_3_) and indirect (*x*_1_ → *x*_3_) causal effects (Gourévich et al., 2006):

(10) $$\begin{array}{l} x_{1}(t)=0.95\sqrt{2}x_{1}(t-1)-0.9025x_{1}(t-2)+e_{1}(t)\\ x_{2}(t)=-0.5x_{1}(t-1)+e_{2}(t)\\ x_{3}(t)=0.5x_{3}(t-1)-0.5x_{2}(t-1)+e_{3}(t) \end{array}$$

where *e*_*i*_(*t*), *i* = [1, 2, 3] is normal noise. A bivariate causality estimator, whether this is linear or nonlinear, cannot distinguish between direct and indirect connections. We expect this to also be the case for the pairwise *C*_*NPMR*_ estimator. We generated 50 different realizations of the model (each of 1000 sample length) and obtained the mean significant causality over all realizations.

Figures 1B–D show the results from linear GC, K-GC and *C*_*NPMR*_ respectively. Connections with causality < 0.005 are not shown, but all significant mean causality values are shown in Supplementary Table 1. Models on the left column are from pairwise/bivariate approach, while the equivalent models from conditional/multivariate approaches are shown on the right column. The indirect connection is incorrectly identified by all pairwise methods (Figure 1, left column). An additional indirect connection is identified by pairwise linear GC and K-GC (*x*_3_ → *x*_2_), but not with pairwise *C*_*NPMR*_. All indirect connections vanish or become negligible for the conditional/multivariate approaches (Figure 1, right column).

The mean sensitivity values for the significant conditional *C*_*NPMR*_ connections are given below:

1. *Q*(*x*_2_ ∕ *x*_1_):
  - *Q*(*x*_2_(*t*) ∕ *x*_1_(*t* − 1)) = 0.194
  - *Q*(*x*_2_(*t*) ∕ *x*_1_(*t* − 2)) = 0.080
  - *Q*(*x*_2_(*t*) ∕ *x*_1_(*t* − 3)) = 0.095
2. *Q*(*x*_3_ ∕ *x*_2_):
  - *Q*(*x*_3_(*t*) ∕ *x*_2_(*t* − 1)) = 0.117
  - *Q*(*x*_3_(*t*) ∕ *x*_2_(*t* − 2)) = 0.079
  - *Q*(*x*_3_(*t*) ∕ *x*_2_(*t* − 3)) = 0.082

The sensitivity indicates that the relationships between *x*_2_ and *x*_1_, and *x*_3_ and *x*_2_ are mainly mediated by the 1-sample lag predictors, *x*_1_(*t* − 1) and *x*_2_(*t* − 1) respectively.

#### 3.1.3. Dataset 3: multivariate mixed coupling model

**Figure 2:**
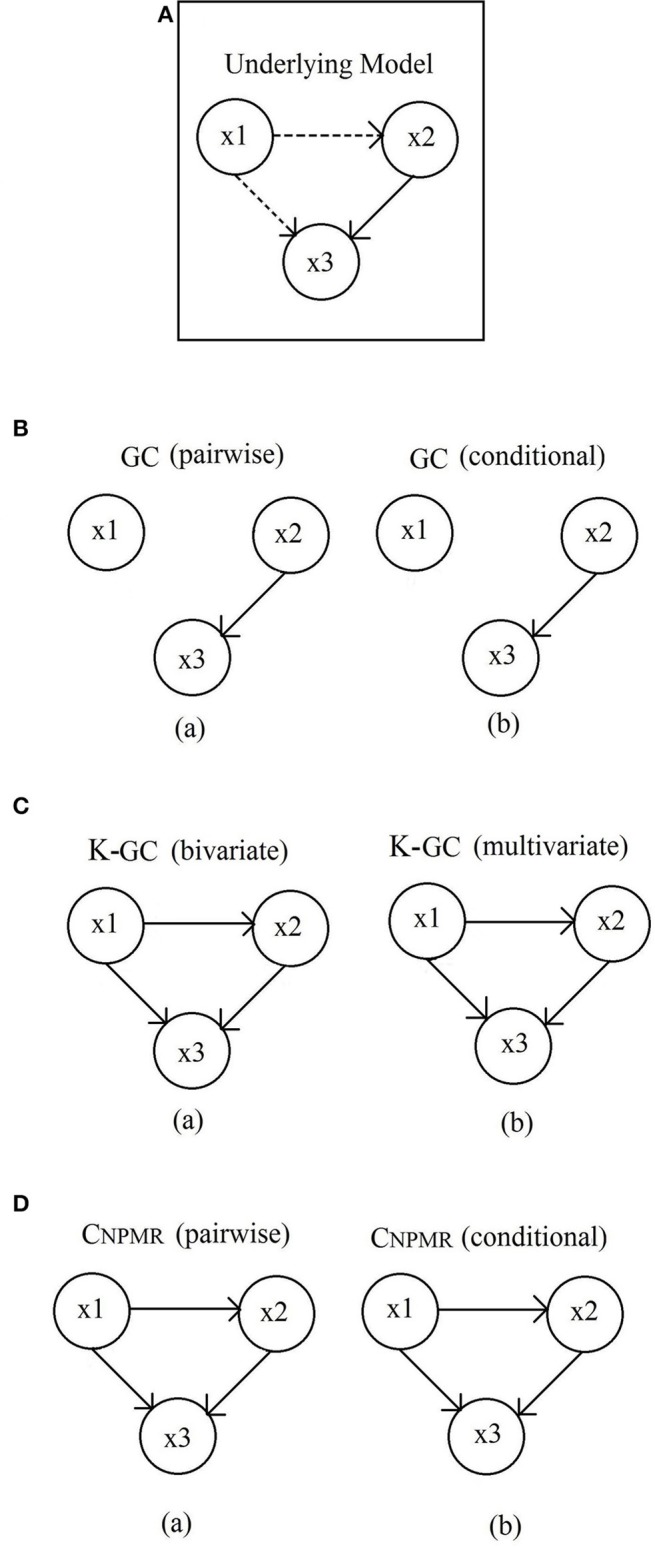
**Dataset 3. (A)** Underlying model. Solid line: linear connection; Dashed line: non-linear connection. **(B)** Linear Granger causality. (a) pairwise *GC*; and (b) conditional *GC*. Unsurprisingly, only the linear connection *x*_2_ → *x*_3_ is identified. **(C)** Kernel-Granger causality. (a) bivariate K-GC; and (b) multivariate K-GC. **(D)** Significant *C*_*NPMR*_ models obtained from (a) pairwise and (b) and conditional method. Both pairwise and conditional *C*_*NPMR*_ estimators identify all linear and nonlinear connections correctly.

This particular model (Figure 2A) has 3 interacting time series with both linear and nonlinear causal effects, while the time series themselves are also nonlinear (Gourévich et al., 2006):

(11) $$\begin{array}{l} x_{1}\left(t\right)=3.4x_{1}\left(t-1\right)\left[1-x_{1}^{2}\left(t-1\right)\right]e^{-x_{1}^{2}\left(t-1\right)}+0.4e_{1}\left(t\right) \end{array}$$

(12) $$\begin{array}{l} x_{2}(t)=3.4x_{2}(t-1)\left[1-x_{2}^{2}(t-1)\right]e^{-x_{2}^{2}(t-1)}\\ \;+0.5x_{1}(t-1)x_{2}(t-1)+0.4e_{2}(t) \end{array}$$

(13) $$\begin{array}{l} x_{3}(t)=3.4x_{3}(t-1)\left[1-x_{3}^{2}(t-1)\right]e^{-x_{3}^{2}(t-1)}\\ \;+0.3x_{2}(t-1)+0.5x_{1}^{2}(t-1)+0.4e_{3}(t) \end{array}$$

where *e*_*i*_(*t*), *i* = [1, 2, 3] is normal noise. Estimations of the mean causalities were performed in a similar manner to previous ones.

Figure 2A shows the underlying model, while the models estimated from pairwise and conditional linear GC, K-GC and *C*_*NPMR*_ are shown in Figures 2B–D respectively. Connections with causality (< 0.005) are not shown, but all mean estimated causality values can be found in Supplementary Table 2. For *C*_*NPMR*_ all connections, both linear and non-linear, are correctly identified. Some negligible but statistically significant connections are also identified. However, these vanish for conditional *C*_*NPMR*_, with the exception of *x*_3_ → *x*_1_ (negligible). For comparison purposes, the corresponding estimates for the pairwise and conditional linear GC, as well as the bivariate and multivariate K-GC estimates. As expected, linear GC only identifies the linear connection *x*_2_ → *x*_3_, while K-GC also identifies the nonlinear connections. Note that K-GC is estimated with σ = 10, as for σ = 1 the estimated values were all 0 (even for normalized data). Negligible but statistically significant (at the 0.05 level) causality is incorrectly identified by K-GC in all directions (univariate and multivariate). Linear GC has only identified the linear connections, as expected. Conditional GC identifies all additional connections as significant, but negligible.

The sensitivity associated with the significant coupling of *C*_*NPMR*_ can be used to infer the underlying structure of the coupling relationships:

1. *Q*(*x*_2_, *x*_1_):
  - *Q*(*x*_2_(*t*), *x*_1_(*t* − 1)) = 0.064
  - *Q*(*x*_2_(*t*), *x*_1_(*t* − 2)) = 0.030
  - *Q*(*x*_2_(*t*), *x*_1_(*t* − 3)) = 0.026
2. *Q*(*x*_3_, *x*_1_):
  - *Q*(*x*_3_(*t*), *x*_1_(*t* − 1)) = 0.068
  - *Q*(*x*_3_(*t*), *x*_1_(*t* − 2)) = 0.029
  - *Q*(*x*_3_(*t*), *x*_1_(*t* − 3)) = 0.025
3. *Q*(*x*_3_, *x*_2_):
  - *Q*(*x*_3_(*t*), *x*_2_(*t* − 1)) = 0.103
  - *Q*(*x*_3_(*t*), *x*_2_(*t* − 2)) = 0.035
  - *Q*(*x*_3_(*t*), *x*_2_(*t* − 3)) = 0.029

#### 3.1.4. Dataset 4: henon maps with variable coupling strength

To study the effect of coupling strength, we apply *C*_*NPMR*_ on unidirectional, non-linearly coupled and non-identical Henon maps with varying coupling strength (Faes et al., 2008a):

(20) $$\begin{array}{l} x\left(t\right)=1.4-x{\left(t-1\right)}^{2}+0.3x\left(t-2\right) \end{array}$$

(21) $$\begin{array}{l} y\left(t\right)=1.4-\left[cx\left(t-1\right)+\left(1-c\right)y\left(t-1\right)\right]y\left(t-1\right)+0.1y\left(t-2\right) \end{array}$$

The system has a non-linear causal effect *x* → *y*, with the degree of coupling controlled by the variable *c*. The specific parameter values were chosen such that the Henon maps operate in a chaotic regime (Faes et al., 2008a). We investigated the behavior of the NPMR-based estimator for increasing coupling strength, *c* = [0, 0.05, 0.1, 0.2, …, 1.0]. For each coupling strength we generated 20,000 samples from the Henon maps and performed non-overlapping windowed estimation of *C*_*NPMR*_ with window length 750 samples. For comparison purposes we also estimated the corresponding Granger causality (GC) and Kernel-Granger causality. The autoregressive model order for the GC was estimated as 6 using the Bayesian Information Criterion (BIC); we used the same embedding dimension (i.e., dependence on past samples) for the *C*_*NPMR*_ estimation (as well as the Kernel-Granger causality estimator).

**Figure 3:**
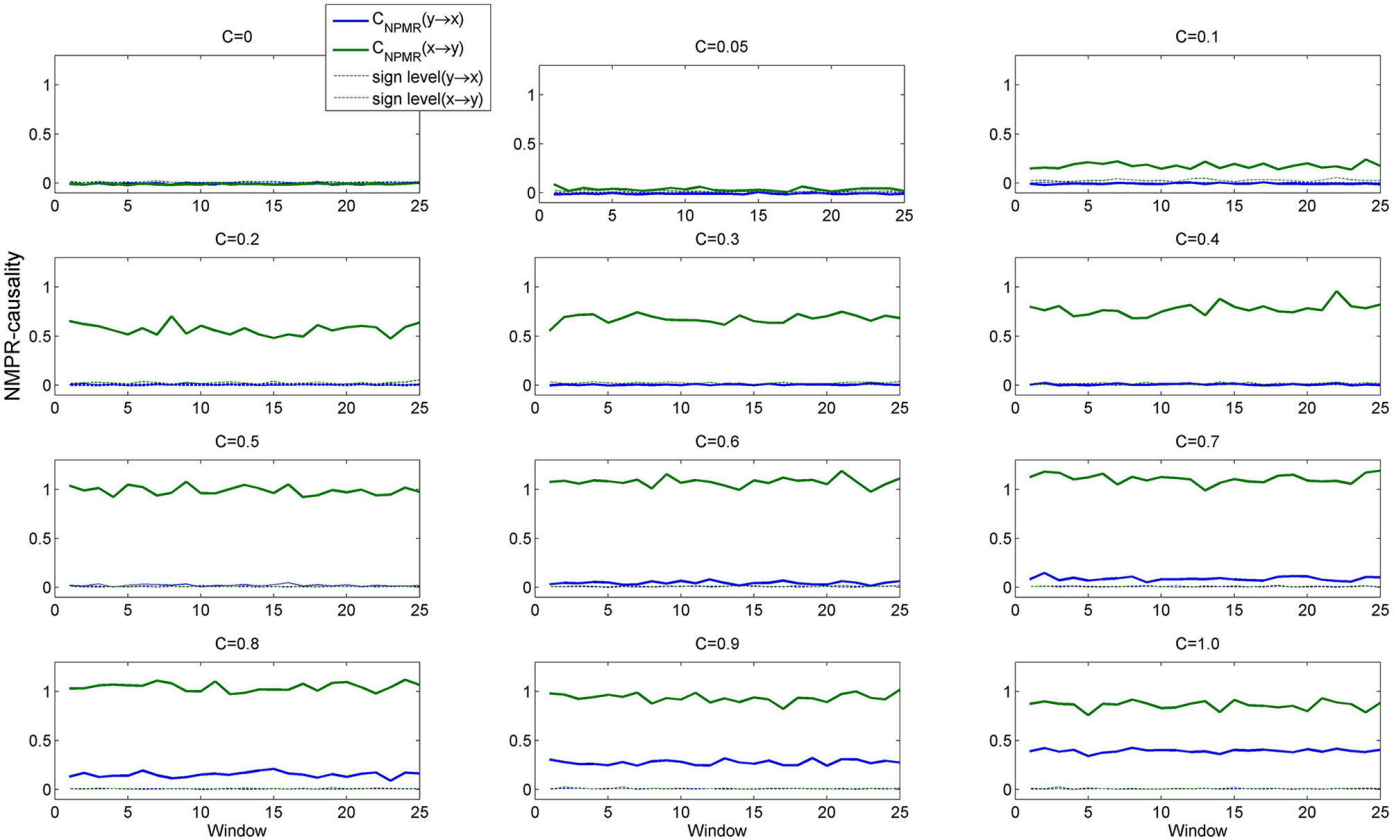
***C*_*NPMR*_ for Dataset 4 (true causal effect *x* → *y*) at coupling strengths *C* = [0, 0.05, 0.1, 0.2, …, 1.0]**. *C*_*NPMR*_(*x* → *y*) is significant for all values of coupling, *c*. *C*_*NPMR*_(*y* → *x*) is only significant for *c* > 0.5, but is always smaller than *C*_*NPMR*_(*x* → *y*).

**Figure 4:**
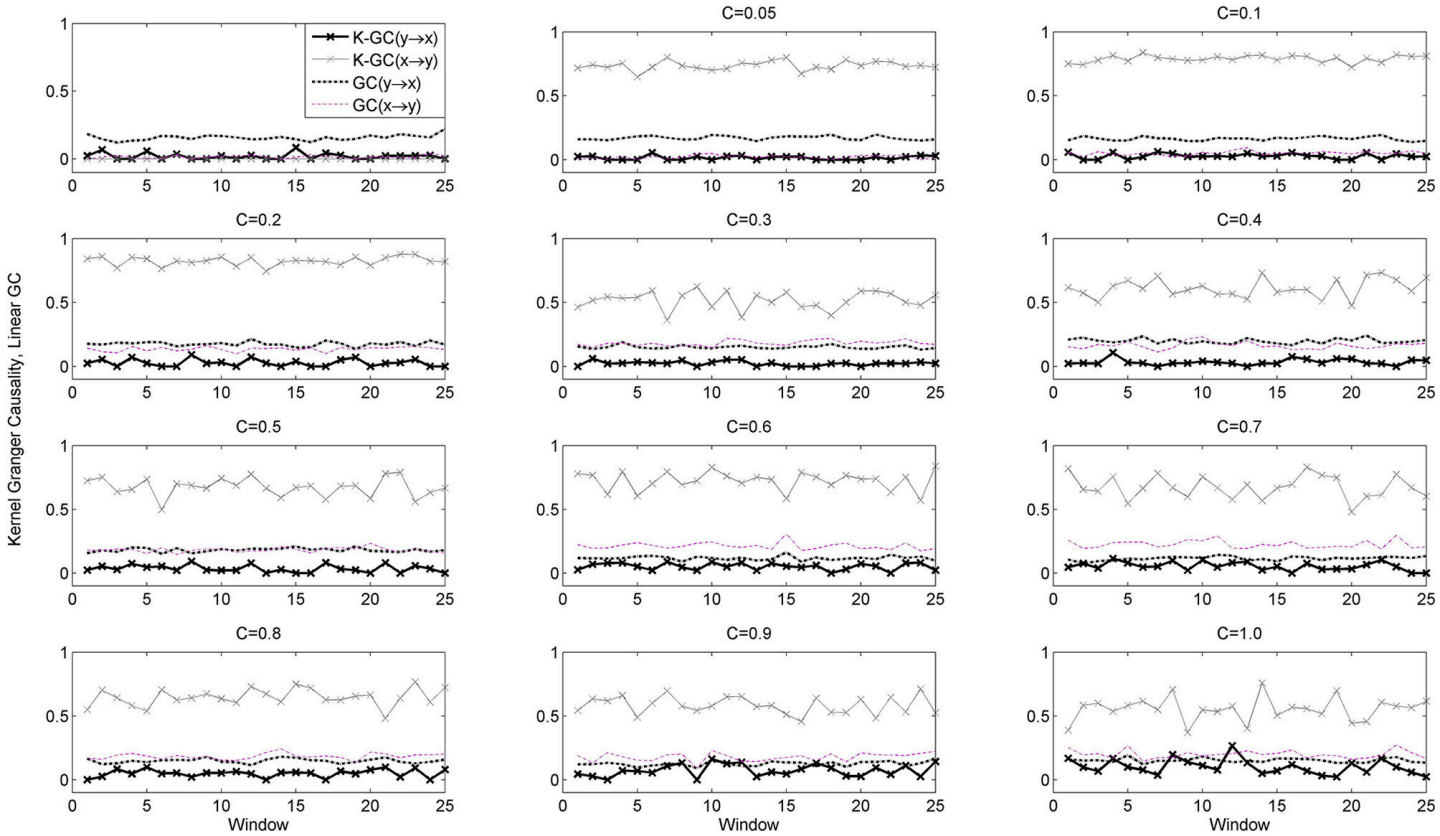
***GC* and K-GC for dataset 4 (true causal effect *x* → *y*) at coupling strengths *c* = [0, 0.05, 0.1, 0.2, …, 1.0]**. K-GC identifies the correct direction of interaction, similarly to *C*_*NPMR*_. *GC*(*x* → *y*) is correctly identified only for *c* > 0.5, while for smaller coupling strengths the opposite causal direction is incorrectly identified.

Figure 3 shows the estimated *C*_*NPMR*_. While *C*_*NPMR*_(*x* → *y*) is significant for all values of *c* > 0, *C*_*NPMR*_(*y* → *x*) also becomes significant for *c* > 0.5 (but always remains below *C*_*NPMR*_(*x* → *y*)). The NPMR-based estimator is able to correctly detect the non-linear causal relationship from *x* to *y* for different coupling strengths. This is in contrast to Granger causality (Figure 4). GC detects the correct causal relationship only for *c* > 0.5. We can deduce from this that GC may be able to detect non-linear coupling when this is large. However, for weaker coupling (*c* < 0.5) linear pairwise GC actually indicates the existence of a causal relationship in the opposite direction, i.e., from *y* to *x*. Kernel-GC also captures the correct direction of interaction but with increased variation in the amplitude of the estimates (Figure 4).

We also observe that *C*_*NPMR*_(*y* → *x*) increases for *c* > 0.5, in contrast to K-GC. On a first assessment this may appear incorrect, as there is no underlying driving in this direction. However, this is related to the increased synchronization between the two Henon maps and has also been reported by Krakovská et al. (2015).

**Figure 5:**
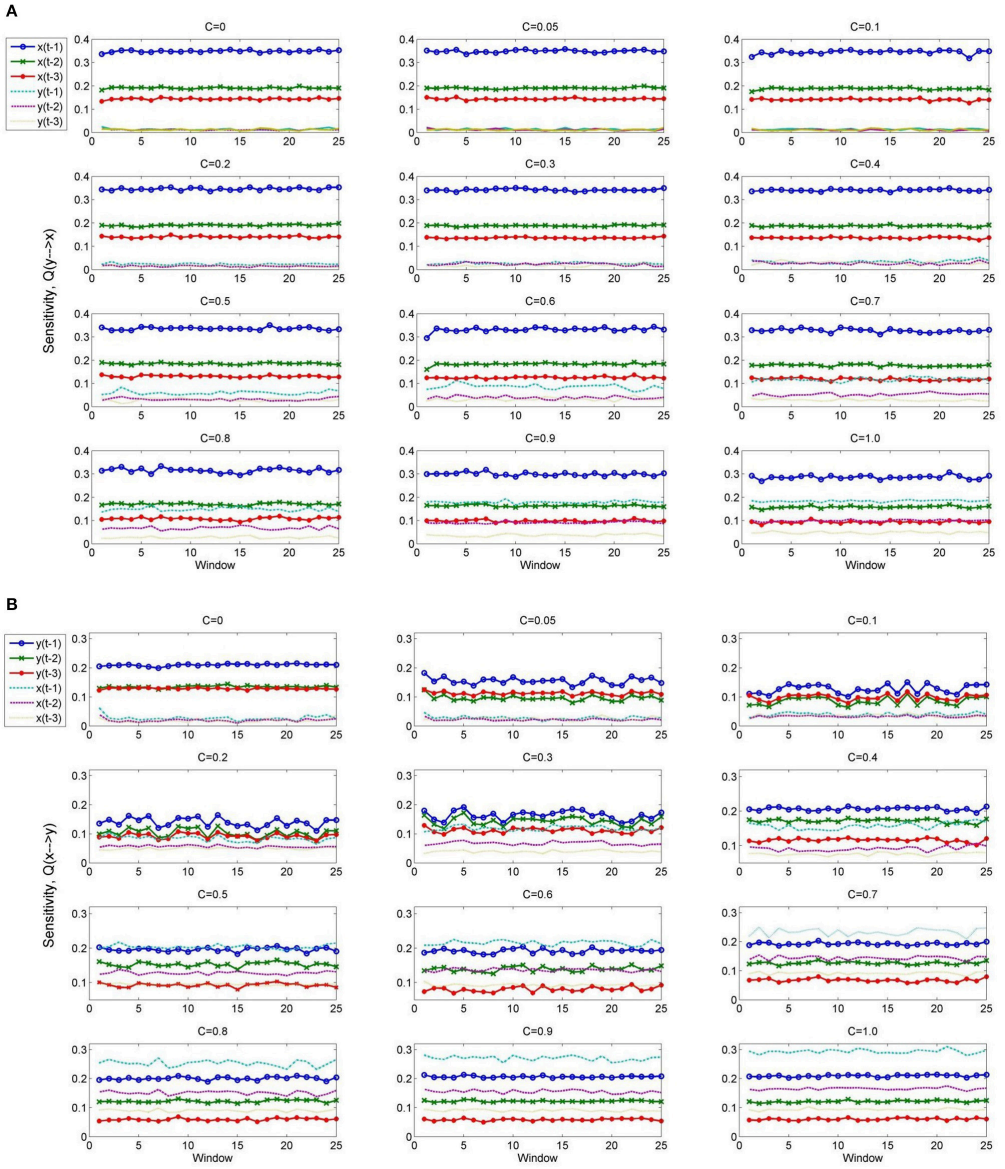
**(A)** Sensitivity, *Q*(*x* ∕ (*x, y*)) for dataset 4 (true causal effect *x* → *y*) at coupling strengths *C* = [0, 0.05, 0.1, 0.2, …, 1.0]. **(B)** Sensitivity, *Q*(*y* ∕ (*y, x*)) for dataset 4 (true causal effect *x* → *y*) at coupling strengths *C* = [0, 0.05, 0.1, 0.2, …, 1.0]. The sensitivity analysis indicates an increasing effect of the past of *x* in the prediction of *y*.

The sensitivity of the *C*_*NPMR*_ estimator provides additional information about the underlying structure of the causal connections. Figure 5A shows the sensitivity of *x* on the past of both *x* and *y*, i.e., *Q*(*x* ∕ (*x, y*)) for values of coupling *c* = [0, 0.05, 0.1, 0.2, …, 1.0]. The sensitivity analysis indicates that the predictors [*x*(*t* − 1), *x*(*t* − 2), *x*(*t* − 3)] are the most important factors in the fitting of *x*. For *c* > 0.6, the sensitivity of *x* to the past of *y*, and particularly *y*(*t* − 1) is increasing. However, *x*(*t* − 1) is the most sensitive predictor for all values of coupling. This corresponds to the correct underlying coupling structure between *x* and *y*. The corresponding sensitivity *Q*(*y* ∕ (*y, x*)) can be seen in Figure 5B. As the coupling increases, *y* starts becoming more sensitive to the past of *x*. For coupling *c* > 0.5, the most important predictor now becomes *x*(*t* − 1), while the past of *x* in fitting of *y* becomes more important overall. For a full coupling at *c* = 1, the predictors are ranked in terms of decreasing sensitivity as follows: *x*(*t* − 1) > *y*(*t* − 1) > *x*(*t* − 2) > *y*(*t* − 2) > *x*(*t* − 3) > *y*(*t* − 3).

#### 3.1.5. Dataset 5: non-linearity via imposing amplitude limits

**Figure 6:**
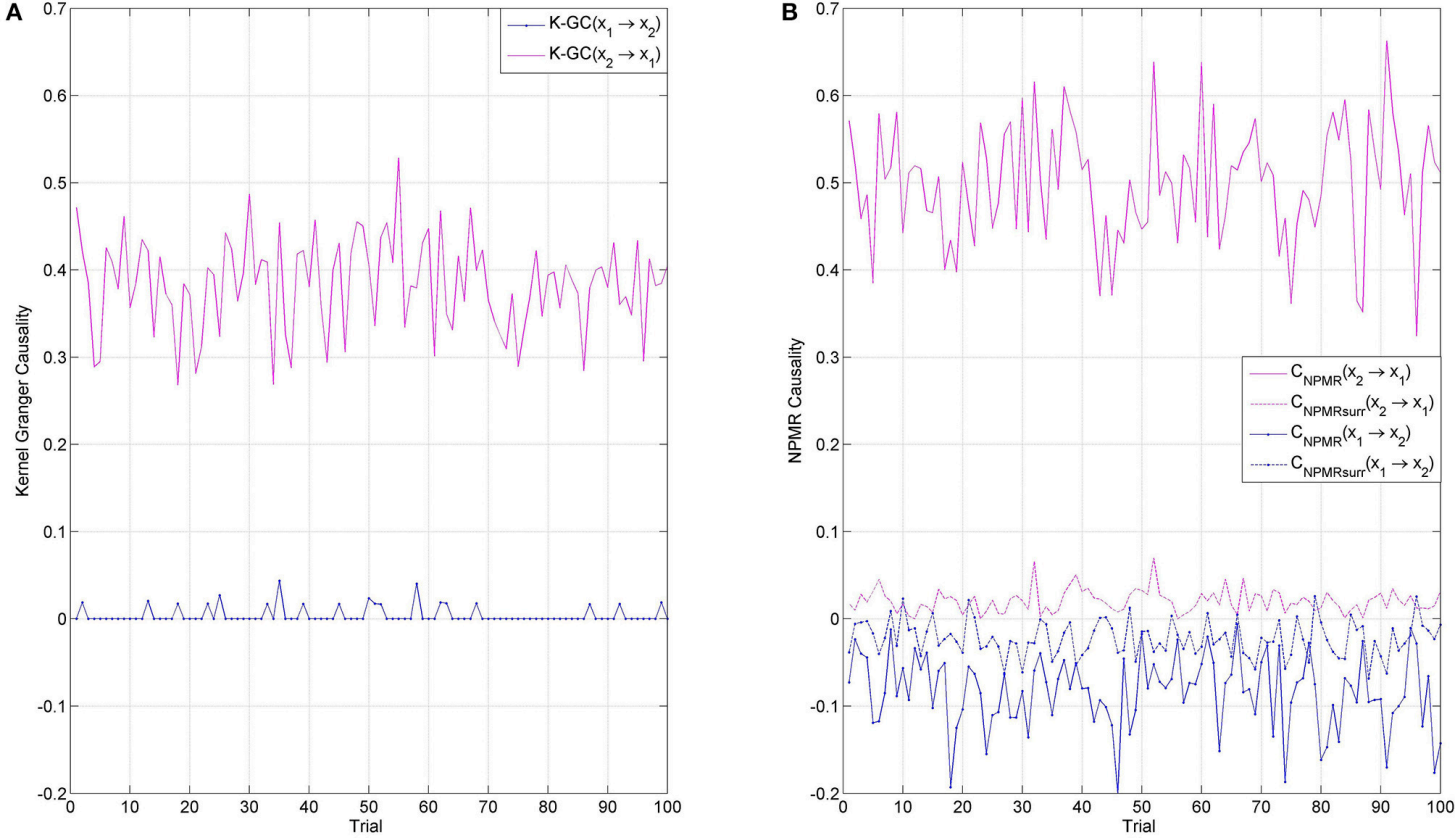
**(A)** Kernel Granger causality (K-GC), and **(B)** *C*_*NPMR*_ for unidirectional nonlinear model (*x*_2_ → *x*_1_) with amplitude limited in the range [0,20]. K-GC shows false causality in the opposite direction (*x*_1_ → *x*_2_) in numerous occassions. This is is contrast to *C*_*NPMR*_, which shows a consistent behavior throughout.

A particular type of nonlinearity that is problematic for Kernel GC is the nonlinearity arising from restricting the time series amplitude to lie within a predefined range. Specifically, the use of a nonlinear kernel in K-GC for this particular application did not lead to performance improvement with respect to the linear kernel. Dataset 5 models this kind of nonlinearity by imposing the limits [0,20] to the amplitude of signals generated using the unidirectional nonlinear model of Equation (9). The limits are imposed in the same manner as described in Marinazzo et al. (2008a), i.e., via the ceiling and floor functions. We generated 100 realizations of this amplitude limited model, with 1000 samples length. Figure 6 shows the performance of the two methods, (a) K-GC and (b) *C*_*NPMR*_. Even though both methods identify the correct direction of information flow (*x*_2_ → *x*_1_), K-GC shows false positive causality in the opposite direction in numerous occassions. This spurious causality is small, albeit significant. In contrast, the same is not observed with *C*_*NPMR*_, which always identifies the direction *x*_1_ → *x*_2_ as non significant. Looking at the sensitivity of *x*_1_ to *x*_2_: (i) *Q*(*x*_1_(*t*) ∕ *x*_2_(*t* − 1)) = 0.0763, (ii) *Q*(*x*_1_(*t*) ∕ *x*_2_(*t* − 2)) = 0.0286, and (iii) *Q*(*x*_1_(*t*) ∕ *x*_2_(*t* − 3)) = 0.015. This indicates that the relationship between the two variables is mainly mediated by *x*_2_(*t* − 1).

### 3.2. Physiological data

#### 3.2.1. Dataset 6: cardiovascular interactions during sleep apnea

The NPMR-based causality estimator was applied on the cardiovascular sleep apnea Santa Fe time series competition dataset (Rigney et al., 1993), available online from PhysioNet (Goldberger et al., 2000). The dataset consists of two files,“b1.txt” and “b2.txt,” that are sequential parts of a single dataset containing approximately 2.4 h each of multivariate data recorded during a sleep study from a patient at the Beth Israel Deaconess Medical Center (Boston, Massachusetts), and sampled at 2 Hz (original sampling rate was 250 Hz). The dataset is a subset extracted from record slp60 of the MIT-BIH Polysomnographic Database (Ichimaru and Moody, 1999) and contains the following 3 parameters: heart rate (H), chest volume (respiration force, R) and blood-oxygen concentration (O). Under normal conditions respiration (R) has a causal effect on the heart rate (H), i.e., *R* → *H*, through the Respiratory Sinus Arrhythmia (RSA) process modulated mainly via parasympathetic activity. The particular dataset is from a patient suffering from sleep apnea, a breathing disorder that affects the normal RSA interaction patterns and feedback mechanisms between heart rate, breathing and blood oxygen concentration, resulting in an unclear relationship between heart rate and breathing. These underlying cardiovascular interaction mechanisms make this dataset an interesting and popular test dataset for causality estimators. It is also interesting for nonlinear causality estimators in particular, as there are nonlinear effects of respiration in the modulation of the heart rate during sleep, which are unaffected by apnea (as opposed to linear effects that are altered in apnea compared to healthy controls; Jo et al., 2007).

Related studies report significant bidirectional causal effects of blood oxygen concentration on respiration and heart rate (Verdes, 2005) and significant bidirectional causality (and transfer entropy) between heart rate and respiration (with *H* → *R* > *R* → *H*) (Schreiber, 2000; Ancona et al., 2004). However, the findings by Ancona et al. (2004), Schreiber (2000), and Marinazzo et al. (2008b) were based on analysis of a 10-min segment from the dataset “b2.txt” (samples 2350–3550). These findings are not representative of the general interaction mechanisms, which vary over time due to the nonstationary nature of the signals involved. Only the analysis reported by Verdes (2005) has been conducted in a windowed manner over the entire record; however, the size of the window used was approximately 10-min (1230 samples), which is unrealistic for physiological signals due to their non-stationary nature.

To test the proposed estimator the two data files, “b1.txt” and “b2.txt,” were concatenated and all three variables normalized to zero mean and unit variance (due to the large differences in heart rate amplitude compared to respiration and blood oxygen concentration). The NMPR-based causality estimator was then applied to the normalized data with embedding dimensions *d* = [3, 4, 5] in non-overlapping windows of *L* = [100, 200, 500] samples.

**Figure 7:**
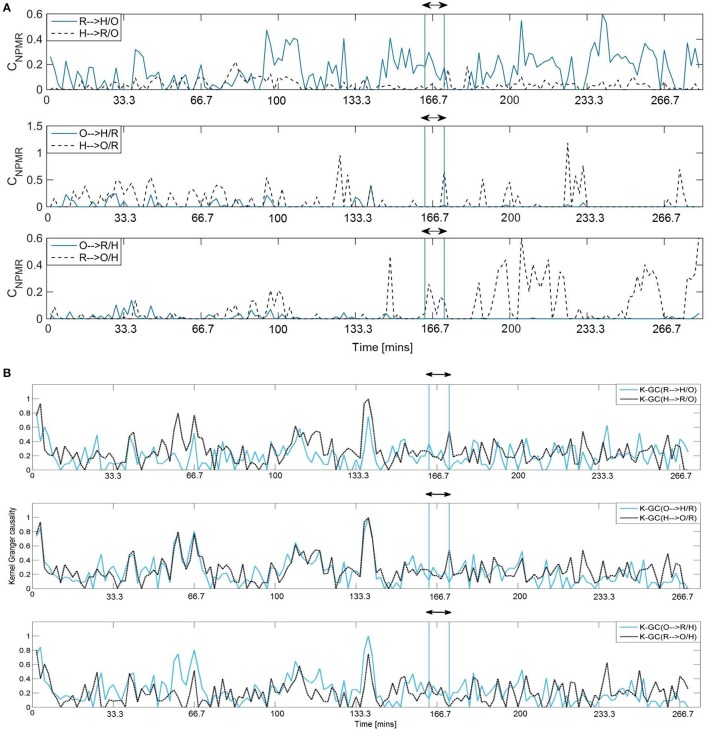
**(A)** Significant conditional *C*_*NPMR*_ estimates for respiration-heart rate (top), blood oxygenation-heart rate (middle) and blood oxygenation-respiration (bottom). The vertical lines around window 100 indicate the 10-min segment analyzed in other studies (see text for details). A strong effect from respiration to heart rate and blood oxygenation is identified. A stronger, but less constant effect from heart rate to blood oxygenation is also identified. **(B)** K-GC estimates, similar as above. It is difficult to identify any particular effect.

Figure 7A shows the estimated conditional *C*_*NPMR*_ for *d* = 4 and *L* = 200. The causal effects identified were independent of the particular combination of *d* and *L* values. A bidirectional causal effect between respiration (R) and heart rate (H) can be identified throughout the record. The interaction *C*_*NPMR*_(*H* → *R* ∕ *O*) is more prominent during the first half of the record and is always smaller than *C*_*NPMR*_(*R* → *H* ∕ *O*). Both respiration and heart rate have a strong significant causal effect on blood oxygenation (*C*_*NPMR*_(*R* → *O* ∕ *H*) and *C*_*NPMR*_(*H* → *O* ∕ *R*)). However, this effect is not simultaneous and manifests itself at different parts of the record. Our main findings (i) agree with Verdes (2005): “respiration is a more decisive factor in determining blood oxygen levels than is heart rate”; and (ii) the physiological mechanism underlying respiration control of heart rate is still present in apnea, as expected from Jo et al. (2007). The NPMR-based estimator also indicates that the RSA, even though prominent, is also somewhat disrupted by sleep apnea, as the causal effect *C*_*NPMR*_(*R* → *H* ∕ *O*) is not constant throughout the record. This is in contrast to the estimates from K-GC (Figure 7B) that do not correspond to any particularly notable causal structure.

**Figure 8:**
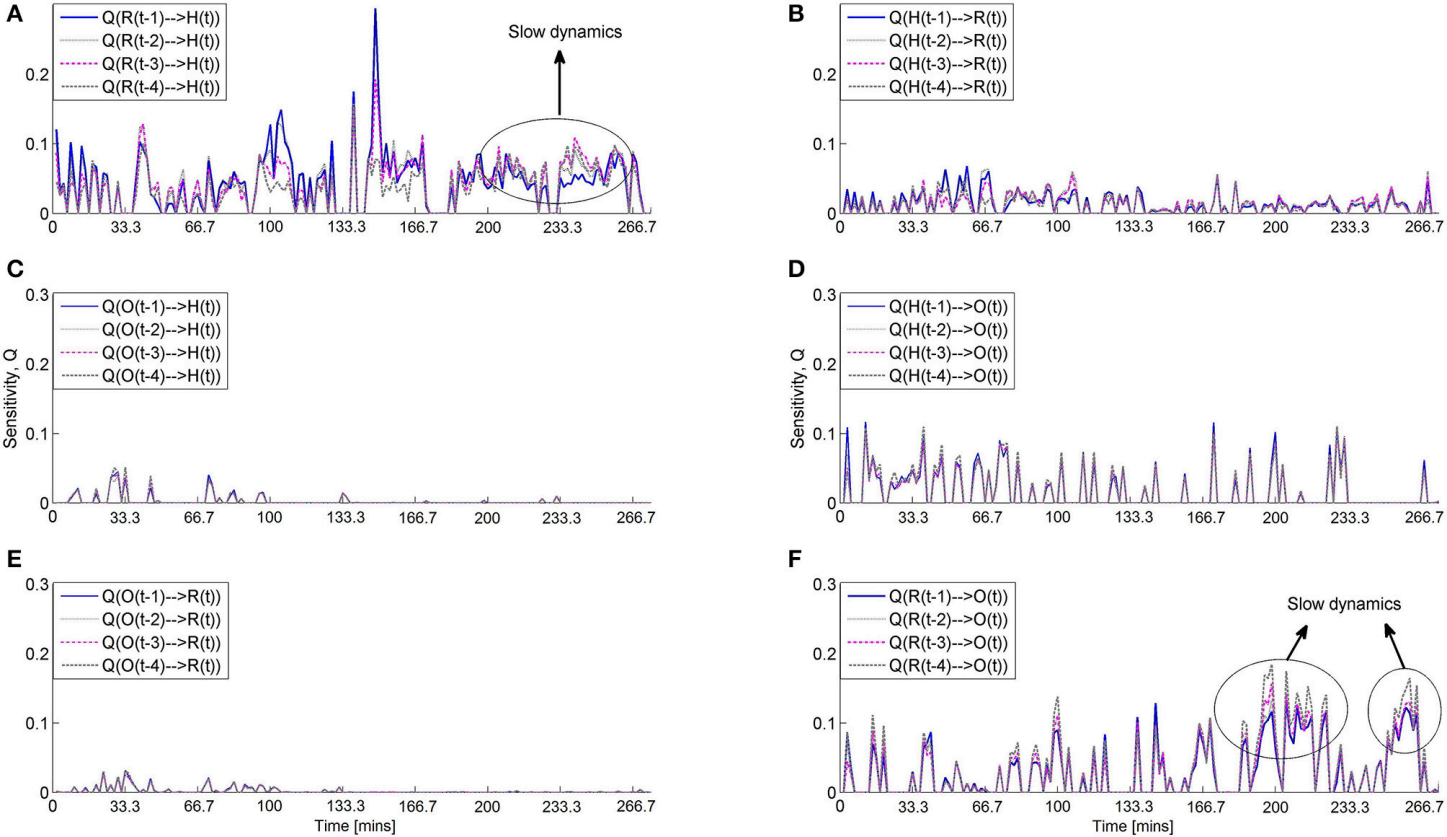
**Significant sensitivity for all possible interactions between heart rate (H), respiration (R) and blood oxygenation (O)**. **(A)** Sensitivity of heart rate to respiration and vice versa **(B)**. **(C)** Sensitivity of heart rate to blood oxygenation and vice versa **(D)**. **(E)** Sensitivity of respiration to blood oxygenation and vice versa **(F)**. The plots indicate a stronger sensitivity of both blood oxygenation and heart rate to values of respiration that are not in the immediate past e.g., time delays 3 and 4.

More information regarding the nature of these interactions is revealed by the sensitivity. Figure 8 shows the sensitivity of the significant multivariate predictions of heart rate, respiration and blood oxygenation. The most interesting plots are the sensitivity of the heart rate to respiration [plot (a)] and the sensitivity of blood oxygenation to respiration [plot (f)]). These plots also support the dynamic nature of the observed relationships and show that the underlying structure is variable over time. At particular points in time, the sensitivity indicates that the relationship between respiration-heart rate and respiration-blood oxygenation is determined by slower dynamics, i.e., time lags 3 and 4 (windows 120–150). This verifies the presence of slow dynamics in the nonlinear effects of respiration on heart, as observed by Jo et al. (2007).

#### 3.2.2. Dataset 7: EEG data during anesthesia

A widespread application of directionality measures is the characterization of changes in brain connectivity during different states. We investigate the behavior of the proposed estimator on electroencephalogram (EEG) activity during the two states of wakefulness and anesthesia. For this purpose we reanalyse a small segment of EEG activity recorded from a volunteer during surgery under general anesthesia. The particular study was approved by the National Bioethics Committee of Cyprus and the patients gave written informed consent for their participation. A description of the full dataset can be found in previous work, e.g., in Nicolaou et al. (2012) and Nicolaou and Georgiou (2014). The specific application is particularly challenging as different connectivity changes during anesthesia are identified depending on which connectivity measure is applied. Causality measures indicate significant increase in interactions during anesthesia, particularly in the fronto-posterior direction (Barrett et al., 2012; Nicolaou et al., 2012). This is in contrast to other methods, which indicate an anesthetic-related decrease in the strength of interactions (Hudetz, 2008; Lee et al., 2009; Ku et al., 2011; Schrouff et al., 2011). An explanation for this discrepancy is yet to be found.

The connectivity changes during wakefulness and propofol anesthesia were investigated using *C*_*NPMR*_ (for comparison purposes Kernel GC was also investigated). The analysis procedure was similar as to that described in Nicolaou and colleagues (please see Nicolaou et al., 2012 for more details). Firstly, the aggregate (average) EEG activity over five predefined areas was obtained as the mean activity over the following areas: left frontal (LF: electrodes Fp1, F7, F3, T3, C3), right frontal (RF: Fp2, F8, F4, C4, T4), left posterior (LP: T5, P3, O1), right posterior (RP: T6, P4, O2), and midline (Z: Fz, Cz, Pz). Secondly, EEG activity corresponding to wakefulness (pre-induction and post-recovery of consciousness) and anesthesia (a 5-minute segment of steady-state and artifact-free EEG activity) was extracted from the continuous record. The two connectivity methods were applied to non-overlapping windows of normalized data (z-score), with window duration 5-s (sampling frequency 256 Hz), and the median *C*_*NPMR*_ and *K* − *GC* values for wakefulness and anesthesia were estimated. The embedding dimension was set to *d* = 6; this was chosen based on previous work, where a 6th order AR model was used (Nicolaou et al., 2012). For *C*_*NPMR*_ we used σ = 1; however, for *K* − *GC* σ = 10 was used, as the estimator did not perform consistently for smaller σ values.

**Figure 9:**
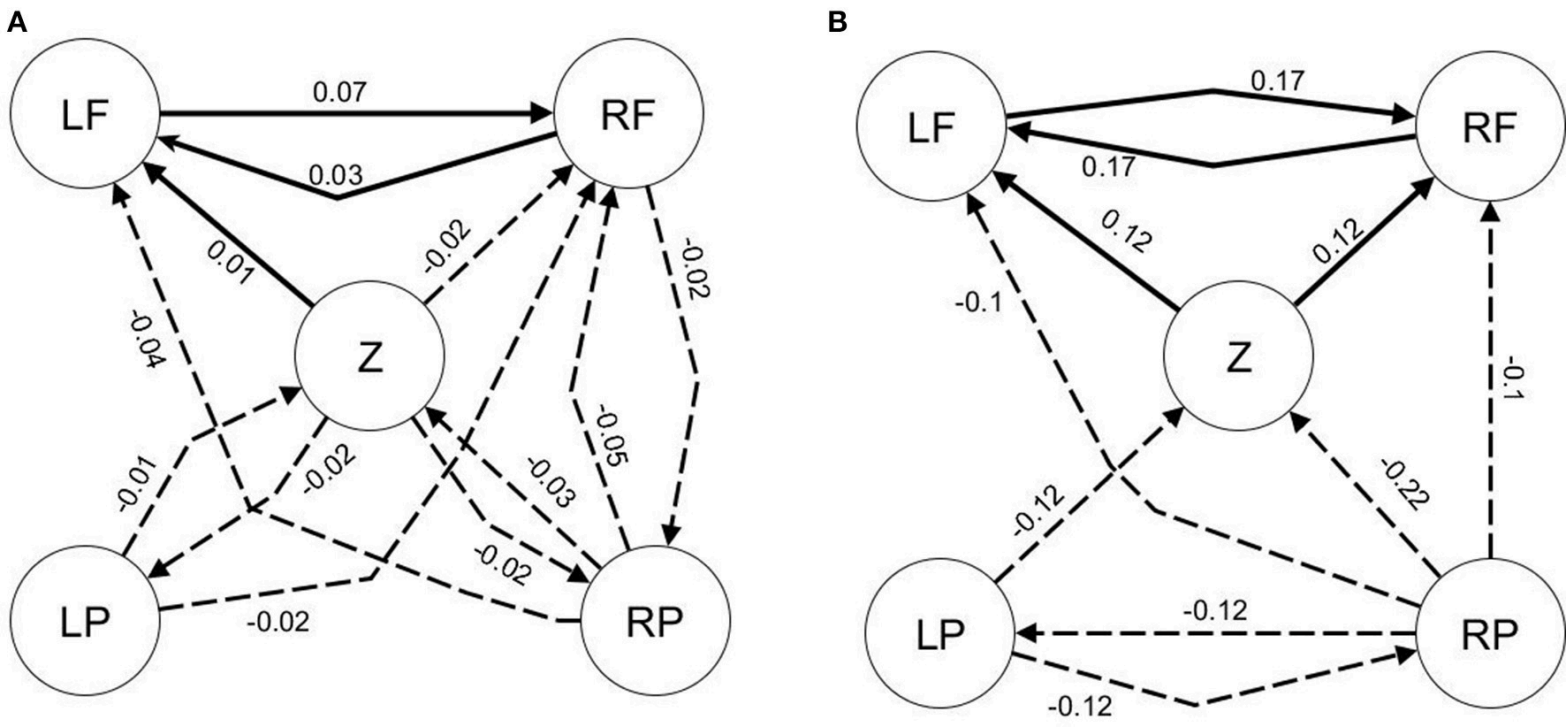
**Changes in connectivity strength between 5 aggregate EEG brain areas from one patient during propofol anesthesia compared to wakefulness for (A) K-GC and (B) *C*_*NPMR*_**. Solid (dashed) lines indicate decrease (increase) of connectivity strength during anesthesia compared to wakefulness. Analysis parameters: (i) embedding dimension *d* = 6, (ii) window length *L* = 1280 samples (5-seconds), (iii) σ: 1 (*C*_*NPMR*_) and 10 (K-GC). Both methods indicate a reduction in fronto-posterior connectivity strength, with K-GC indicating more bi-directional changes. *C*_*NPMR*_ is more consistent with literature and findings from other methods, whereby a reduction in unidirectional connectivity strength from posterior to frontal areas is identified.

Figure 9 shows the resulting differences in median connectivity values between the five aggregate areas for K-GC (left) and *C*_*NPMR*_ (right). For visualization purposes the strongest connectivity changes are shown (*K* − *GC* > |0.01| and *C*_*NPMR*_ > |0.1|). Solid (dashed) lines indicate an increase (decrease) of connectivity strength during anesthesia compared to wakefulness. *C*_*NPMR*_ shows a unidirectional reduction of connectivity from posterior to frontal areas, which is consistent with the literature and findings from other methods. K-GC indicates bidirectional connectivity changes, which is not fully consistent with the literature, while the connectivity strength is almost negligible. An interesting observation is that while the findings of *C*_*NPMR*_ agree with findings from other methods reported in the literature, they are contradictory to those from linear GC. Recalling similar contradictory observations from Henon maps with variable coupling strength (dataset 4), one possible explanation is the existence of weak nonlinear relationships in the brain network that result in linear GC indicating an opposite direction of causality.

**Figure 10:**
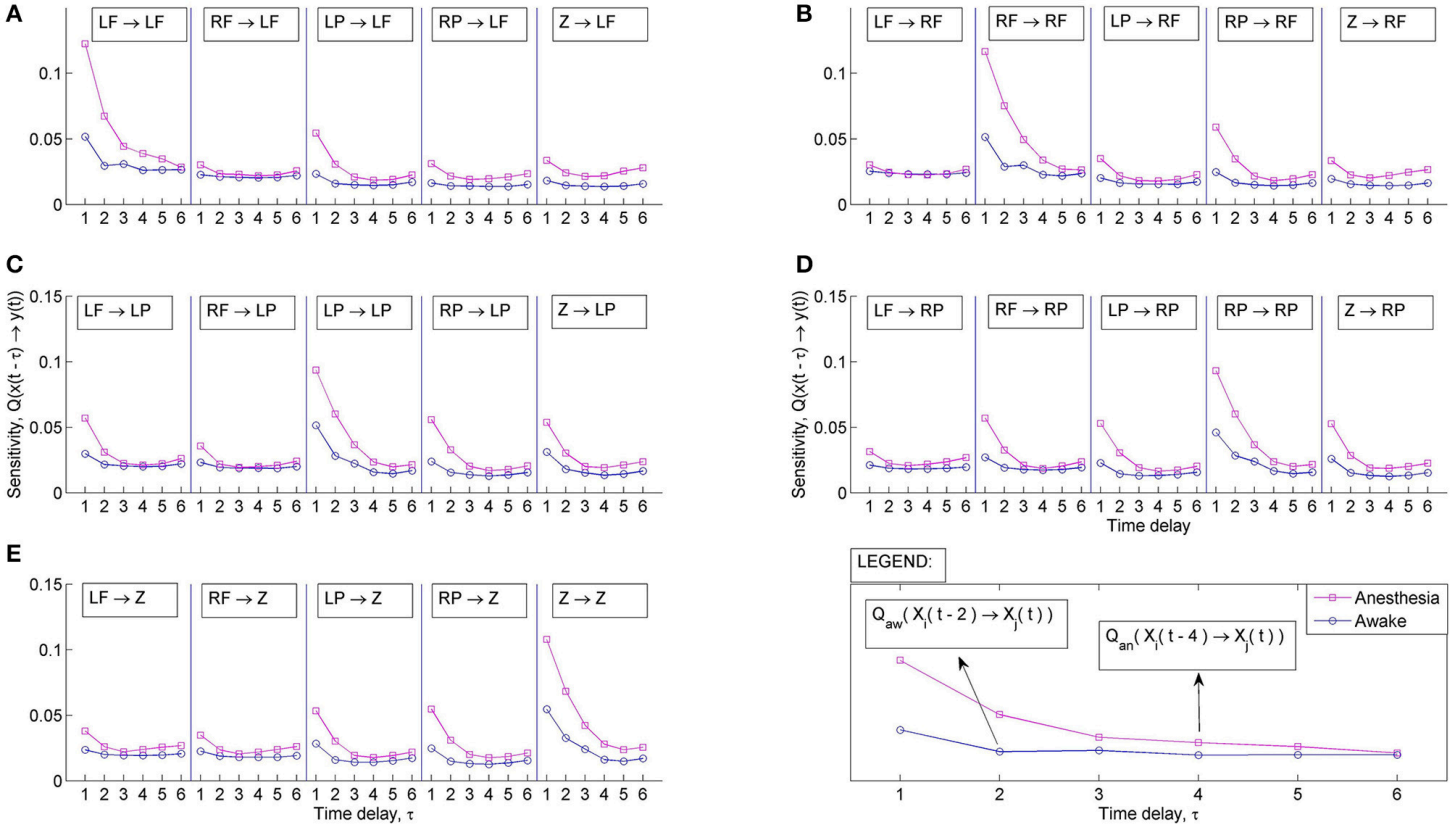
**Sensitivity of prediction between the five EEG aggregate areas during wakefulness and anesthesia**. Sensitivity from all other areas to: **(A)** Left Frontal (LF). **(B)** Right Frontal (RF). **(C)** Left Posterior (LP). **(D)** Right Posterior (RP). **(E)** Midline (Z).

Figure 10 shows the mean sensitivity for all multivariate predictions during wakefulness and anesthesia. A general increase of sensitivity is associated with the anesthetic state. This increase is more prominent for lags 1–2 and 5–6, and between posterior, posterior-midline and ipsilateral posterior-frontal areas. The sensitivity indicates that during anesthesia the response variable is more sensitive to changes in the immediate past, but is also influenced by changes that are even further in the past. Therefore, the underlying models of interaction are more complex than a simple 1st order relationship and the entire system is more sensitive to slow dynamics during anesthesia. A full physiological interpretation of the information obtained from sensitivity could be obtained by combining this with existing models of EEG generation during wakefulness and anesthesia (e.g., the models by Steyn-Ross et al., 2004 and Bojak and Liley, 2005), and seeing how the sensitivity ties in with, or differs from, these models.

## 4. Discussion

Our results support that the use of Nonparametric Multiplicative Regression (NPMR) is appropriate for causality estimation and estimates of pairwise or multivariate causality can be obtained directly and without any modification of the method. *C*_*NPMR*_ captures the true linear and nonlinear relationships in artificially generated time series, as well as connectivity changes in two physiological datasets (cardiovascular interaction during sleep apnea, and brain interaction during wakefulness and anesthesia). The proposed method works well for a diverse set of problems. This versatility is partly due to the non-parametric nature of the method, but also due to the property of NPMR to detect (in theory) any function that is a smooth mapping between a response variable and a set of predictors, without any restriction in terms of its form (McCune, 2011); some examples of the surfaces that can be captured by NPMR can be found in Lintz et al. (2011). This inherent property of NPMR could also be considered as a limitation, however, as NPMR cannot accurately describe surfaces that have discontinuities or cusps. Another factor behind the versatility of the proposed method is the use of phase space reconstruction from multiple observed time series as a means of incorporating past information in the prediction: the dynamics of the entire system can be studied simply by studying the dynamics of the phase space (embedded time series). As long as an appropriate embedding dimension is used, then the phase space reconstruction can uncover structure from what appears to be random time series data of single predictor variables, leading to the formation of more accurate models of real-world dynamics. It is also possible to use different embedding dimensions for each predictor, depending on the individual predictor dynamics. In conjunction to this, the influence of each individual predictor can be controlled via the tolerance of the distance function. The investigations performed in this study used the same tolerance value for all predictors (σ = 1) as a proof-of-principle. It is, however, advisable that the tolerance is optimized for each individual predictor. This could mean placing more/less importance on samples that are nearer/further in the past. The ability to perform such fine tuning of how past information is used in the prediction is an additional factor of creating more accurate models. Regarding the use of the Gaussian distance function, a limitation of it is that a zero weight can be obtained for signals that have large amplitude. This results in non arithmetic estimates, which affect both the *C*_*NPMR*_ and sensitivity values. In these cases it is advisable to first normalize the signals.

### 4.1. Related methods

The nonparametric multiplicative regression that replaces AR modeling has some similarities to other nonparametric techniques that use smoothing functions, such as Radial Basis Functions (RBFs) - when a Gaussian weight is used in NPMR - and General Additive Models (GAMs). NPMR is in fact a special case of a GAM (Schulz and Childers, 2011), with the main difference lying in the way individual factors are combined. In GAMs, individual factors are combined additively, while in NPMR the response is modeled multiplicatively, i.e., in a way that allows for the effect of each variable to depend on the value of another variable (Potapova and Winter, 2006). Considering RBFs, the difference is somewhat less intuitive. At a first glance, the product of the NPMR weights (Section 2.1, Equation 3) reduces to a single exponential, with the sum of Euclidean distances in the exponent. This is similar to RBF network nodes and this simplification of the multiplicative weights only occurs when a Gaussian kernel is used. It is not necessary that the conversion of an exponential function to an additive function is performed in NPMR, as another weighting function that is not exponential can be used. However, in an RBF setting the response variable is estimated as the sum of the weighted distances of each predictor from the response variable (or, more commonly, from a representative cluster center), where the weights have to be estimated via some training procedure. In contrast, in NPMR the distances are the actual weights and these are combined multiplicatively to determine the influence of each point of the time series in the prediction.

**Figure 11:**
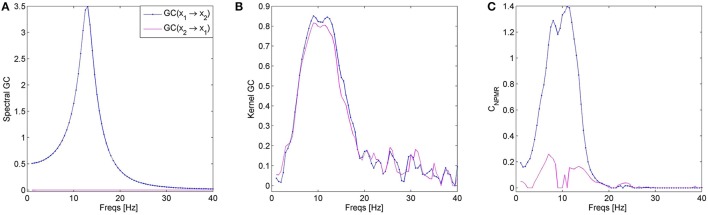
**Bandpass filtering as a means of estimating spectral causality. (A)** Spectral GC estimated from Geweke's method for data *x*_1_ and *x*_2_ from dataset 3. **(B)** Kernel-GC and **(C)** *C*_*NPMR*_ estimated from bandpass filtered data. Even though some smearing is observed, bandpass filtering could be used to obtain spectral causality from *C*_*NPMR*_, but not from K-GC.

Another related technique is Kernel-Granger Causality (K-GC). However, the proposed method is not equivalent to K-GC, as is also evident from the differences in their behavior. The two methods are distinct and any similarity between them only arises from the use of the same kernel function, for example when a Gaussian kernel is used in both methods. The main difference is that K-GC transforms the data in the feature space of suitable kernel functions where linear Granger causality is then performed via regression. In *C*_*NPMR*_, however, the data is not transformed into a kernel feature space and linear regression is not performed. Kernel functions are simply used as a means of estimating the influence, i.e., the weight, of the predictor points in the estimation of a target point. The proposed method is purely data driven and the estimated global model does not have any particular form. The relationship between K-GC and its linear regression basis is also evident from the fact that K-GC is more affected by bandpass filtering (Figure 11), just like all regression-based causality methods (Florin et al., 2010). On the contrary, *C*_*NPMR*_ is not affected by filtering, and this could also be used as the basis for estimating frequency domain causality. Another difference is that *C*_*NPMR*_ can take into account new data points as these become available, without having to re-calibrate the method. This is not possible with K-GC (and in fact with the majority of available causality estimators), as re-estimation of the cluster centers would be required for every new data point.

### 4.2. Additional considerations

**Figure 12:**
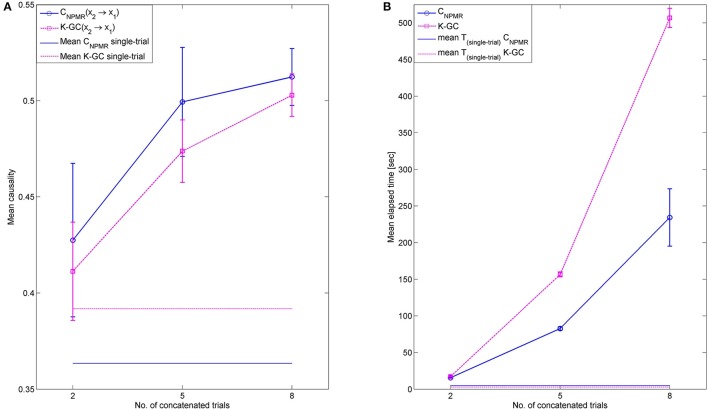
**(A)** Mean (and standard deviation) of multi-trial causality for *C*_*NPMR*_ and K-GC as a function of the number of concatenated trials. Results averaged over 10 repetitions with randomly chosen concatenated trials. Horizontal lines show the mean causality when a model is fit to each trial independently. **(B)** The corresponding mean (and standard deviation) of elapsed time (in seconds). *C*_*NPMR*_ is more computationally efficient than K-GC for larger sample sizes.

1. *Choice of kernel tolerance*. The kernel tolerance in NPMR is equivalent to the smoothing parameter or bandwidth in a general statistical sense. One of the advantages of NPMR is that it allows predictors to have a separate tolerance and, thus, importance in the subsequent prediction. The weight given to a particular observation is related to its distance from the target and this relationship is defined by the chosen kernel. A Gaussian kernel centered at the target point provides a simple and flexible way of defining this distance-based weight, with the tolerance (standard deviation, σ) defining how rapidly the weights dimish with distance from the target (McCune, 2011). The kernel tolerance is a parameter that should be optimized for the particular application and for different predictors and this can be achieved through free search or tuning (McCune, 2011). Since the focus of the particular study is to provide a proof-of-principle for NPMR-based causality estimation, we did not optimize the kernel tolerance for the datasets or predictors individually. However, we perform some preliminary investigation of how the tolerance may affect the estimated causality. Supplementary Figure 1 shows an example from causality estimation for Henon maps (dataset 4) for different values of σ. Estimates obtained with smaller values of σ exhibit larger variance; however, the patterns of estimated causality agree with the ground truth for all tolerances investigated. Despite this, optimization of the tolerance is recommended and doing so based on the data characteristics and without resorting to free search remains the subject of future work.
2. *Embedding delay and dimension*. A number of methods have been proposed in the literature for estimating an appropriate time delay and embedding dimension. The time delay can be estimated as the first minimum of the mutual information (Fraser and Swinney, 1986) or the time when the autocorrelation function approaches 1/e. More emphasis is given on the embedding dimension (e.g., Kennel et al., 1992; Cao, 1997). More information regarding the various estimation techniques and their practical application can be found in Galka (2000). Any of these methods could be used to select the appropriate delay/embedding dimension for the individual predictors; however, the method by Cao is the most popular for embedding dimension estimation. To see how *C*_*NPMR*_ could be affected by over/under-estimation of the embedding dimension we performed preliminary investigations with Henon maps (dataset 4) and estimated causality while varying the embedding dimension *d* = 1, 2, 3, 4, 6, 8. Supplementary Figure 2 shows the estimated causality for the generated Henon maps. Our initial findings indicate that larger embedding dimension may lead to smaller amplitude. Even though the choice of embedding dimension does not seem to affect the pattern of causality, the appropriate embedding dimension should first be estimated and used in subsequent analysis.
3. *Frequency domain causality and filtering*. The use of bandpass filtering as a means of estimating spectral causality is not suitable for AR-based GC methods, as filtering modifies the smoothness of the data, resulting in large increase in the required model and incorrect results (Florin et al., 2010). Spectral causality is, thus, obtained via a method proposed by Geweke, which is similar to performing a Fourier transform of the estimated autoregressive coefficients at specified frequencies, with frequency resolution specified by the model order (more information on frequency domain causality can be found in Cohen, 2014). Since NPMR does not have specific model parameters, Geweke's approach cannot be applied here. However, the lack of parameters is also an advantage in this respect as it might be possible to both apply filtering without affecting the underlying causality and obtaining a spectral representation of causality by bandpass filtering the data and applying *C*_*NPMR*_ to the filtered data. This would be an important advantage of *C*_*NPMR*_. Even though the focus of this study is to introduce the use of NPMR-based modeling for causality estimation, we perform a preliminary investigation of whether bandpass filtering could be used as a means of obtaining spectral causality without distorting the underlying relationships. We generate a random realization of variables *x*_1_ and *x*_2_ from Dataset 3 (underlying relationship: *x*_1_ → *x*_2_) with sampling frequency *fs* = 100 Hz and length 1000 samples. We estimated the spectral GC using the Matlab® function “*pwcausal*” from the BSMART toolbox (Cui et al., 2008). This revealed that the effect *x*_1_ → *x*_2_ occurs at approximately 10-18 Hz (Figure 11A). We then bandpass the signals with an FIR filter (Matlab® function “*fir1*,” filter order 50, Hamming window) and estimate the significant causality using *C*_*NPMR*_ and K-GC, at the frequency range 1–40 Hz (with 0.5 Hz resolution and bandpass bandwidth 5 Hz). Figure 11 shows the estimated causality for the bandpass filtered signals with (b) K-GC and (c) *C*_*NPMR*_. Despite some smearing, which could also be due to the 0.5 Hz filter resolution, and some significant causality in the opposite direction (*x*_2_ → *x*_1_), *C*_*NPMR*_ captures the underlying causal relationship *x*_1_ → *x*_2_ at the expected frequency range. This supports the feasibility of using bandpass filtering with *C*_*NPMR*_ as a means of estimating causality in the frequency domain. On the contrary, this is not possible with K-GC (Figure 11B); this is expected as K-GC is still based on regression in the kernel space and would be affected by filtering. Even though these are only preliminary findings, they are highly encouraging.
4. *Multi-trial data.* It is common in neuroscience applications that data from multiple trials are available for analysis. To estimate causality of multi-trial data one can either fit a model on data from each trial independently and obtain an average measure of causality or concatenate the multi-trial data and fit a single model. Neither approach is ideal; the former involves fitting a large number of models, thus increasing computational cost, while the latter will likely lead to data that breaks the stationarity assumption (essential for the majority of causality methods) while also increasing computational complexity (due to the very large size of the concatenated data). These are general problems that also apply to the proposed estimator, with the exception of non-stationarity as *C*_*NPMR*_ does not require the data to be stationary. Even though how to best deal with multi-trial data using *C*_*NPMR*_ is not within the scope of this study, we conducted some preliminary investigations. We generate 15 trials from Dataset 1 (nonlinear causality from *x*_2_ → *x*_1_), each of length 1000 samples. As a compromise between model fit and computational complexity, we repeat the concatenation process *B* = 10 times using a randomly chosen subset of *N*_*tr*_ trials each time, estimate the NPMR model and obtain the average causality (concatenated causality). We also applied a model to each of the 15 trials independently and obtained the mean causality over all trials (multi-trial causality). For comparison purposes we also applied Kernel-GC in a similar manner. For both methods we used an embedding dimension *d* = 3 and tolerance σ = 1. Figure 12 shows the results for *N*_*tr*_ = [2, 5, 8]. For both methods the concatenated causality corresponded to the true causal direction; however, causality was inflated when compared to the averaged multi-trial causality. The inflation increased when more trials were concatenated. The run time (for *C*_*NPMR*_ this also includes the time to perform surrogate analysis), measured using the Matlab® functions *tic* and *toc*, is also shown in Figure 12. As expected, run time increases when more trials are concatenated, with *C*_*NPMR*_ being substantially more computationally efficient than Kernel-GC, particularly for larger sample sizes. We also note here that a multi-trial implementation of K-GC, where trial concatenation is performed as part of the implementation, is also available in the K-GC toolbox. The results from this multi-trial implementation were similar.
5. *Signal-dependent noise model.* The main class of artificial models used in causality-related investigations assume time invariant covariance structure and normally distributed noise that is independent of the signal. Recently, Luo and colleagues proposed a signal-dependent noise causality model and a likelihood ratio test for causal inference (Luo et al., 2013a). This type of signal-dependent noise can be found in some physiological time series, e.g., fMRI BOLD time series. Causality algorithms fail to capture any causal relationships arising from such signal-driven noise interactions and this was an interesting test for *C*_*NPMR*_. We performed preliminary investigations using the first-order Autoregressive Baba-Engle-Kraft-Kroner (AR-BEKK) model for two univariate time series described in Luo et al. (2013a) (see Supplementary Material Appendix 3 for the mathematical description of the model). In this model one can control for the presence of signal-dependent noise causal influence through a set of parameters associated with the model variance. We generated 100 realizations (length 1000 samples) of this model by adapting the function *arma_bekk_simulate.m* from the Granger causality estimation toolbox by Luo et al. (2013b), 50 with causal influence *y*_*t*_ → *x*_*t*_ and 50 with causal influence *x*_*t*_ → *y*_*t*_, and estimated *C*_*NPMR*_ (parameters: σ_*j*_ = 1, *d* = 3) and K-GC (parameters: Gaussian kernel with radius 1, model order 3). Our preliminary investigations indicate that neither method was able to consistently recover the correct causal structure, with K-GC also showing significant causal influences in the opposite directions. However, we cannot conclude with certainty that *C*_*NPMR*_ is unsuitable for such signals. It is possible that *C*_*NPMR*_ performance could be improved after optimizing for the tolerance, embedding and delay parameters.

### 4.3. Guidelines for application to real data

Below are some general guidelines for the application of *C*_*NPMR*_ to neuroscience data:

1. *Normalization*. Normalization is usually good practice and is necessary when the different time series have large differences in amplitude. Even though it was not necessary to normalize the artificial data, we did normalize (z-score) the physiological data and we do recommend this as good practice.
2. *Filtering*. It is generally a good approach to apply a high-pass filter, with high pass between 0.1 and 0.5 Hz, to minimize any slow drifts. A notch filter at 50 Hz (or 60 Hz) is also recommended to attenuate electrical noise. Since *C*_*NPMR*_ does not appear to be as susceptible to filtering as other causality methods, such filtering is recommended and it may be possible to use bandpass filtering as a means of estimating causality in the frequency domain. High-pass filtering should be applied to continuous data prior to any segmentation, to avoid window edge artifacts that could affect the accuracy of the connectivity analysis.
3. *Window size*. The amount of data in a segment is a general concern of windowed connectivity analysis, with both advantages and disadvantages of shorter and longer windows (Cohen, 2014). Longer windows give estimates that are more stable, but they may be non-stationary and it may be more difficult to isolate task-related connectivity; particularly, when connectivity occurs at a time frame that is much shorter than the window length it is possible that this is not detected due to averaging with a much longer task-unrelated segment. On the other hand, shorter windows are more likely to be stationary and more sensitive to transient events, but the fitted model may not be as accurate. As NPMR is a non-parametric data-driven method, it is less affected by issues of stationarity and model parameter estimation, and the most important consideration is to use enough data such that the task-related dynamics are captured. However, as the window size increases, so does the computational cost. In neuroscience applications window length varies from ~ 100 ms to a few seconds, and we recommend that a similar window size is also used for *C*_*NPMR*_.
4. *Time delay embedding parameters*. In terms of the individual predictors, the choice of embedding dimension affects the amplitude of the estimated causality, but not the pattern of underlying causal relationships. Despite this, we recommend that the appropriate embedding parameters are estimated using any of the methods proposed in the literature. Using an appropriate time delay would also allow longer dynamics to be taken into account in the prediction of the current sample. In terms of the NPMR model as a whole, McCune (2011) recommends a procedure for assessing the model fit via the “cross *R*^2^” (Equation 8). The model fit could be assessed while new predictors (i.e., more embedding dimensions) are added and a threshold in model fit improvement could be used to determine whether inclusion of the additional predictor is worthwhile.
5. *Number of sensors*. The complexity imposed by the number of sensors is a general problem for multivariate methods, and the proposed method is no exception. A potential solution could be to apply a dimensionality reduction method first, such as Principal Component Analysis, and then apply the method to the reduced sensor space.

## 5. Conclusions

We present a nonparametric estimator of causality, *C*_*NPMR*_, that uses Nonparametric Multiplicative Regression (NPMR). The main NPMR technique, which was first introduced for habitat modeling, was modified such that past information of the time series is included in the modeling. The method has been demonstrated on artificial data with linear and nonlinear causal relationships, as well as on physiological data. *C*_*NPMR*_ addresses many of the limitations associated with linear Granger causality, as well as other nonlinear causality estimators proposed in the literature. Its nonpametric nature and its ability to capture nonlinear relationships make it appealing for a number of applications.

## Author contributions

NN: conception and design of the work; analysis of data; drafting of work. TC: critical revising of work. NN, TC: interpretation of data; final approval of published version.

## Funding

This research leading to these results has received funding from the People Programme (Marie Curie Actions) of the European Union's Seventh Framework Programme (FP7/2007-2013) under REA grant agreement n^*o*^ 623767.

### Conflict of interest statement

The authors declare that the research was conducted in the absence of any commercial or financial relationships that could be construed as a potential conflict of interest.
