# Supplementary material for: A Nonlinear Causality Estimator Based on Non-Parametric Multiplicative Regression
Source: Front Neuroinform. 2016 Jun 14;10:19. doi: 10.3389/fninf.2016.00019 (PMC4905976; doi:10.3389/fninf.2016.00019)
Supplement: Supplementary file 1 [file Table1.PDF]

Supplementary Material: A nonlinear causality estimator based on Non-Parametric Multiplicative Regression

Nicoletta Nicolaou * and Timothy Constandinou

*Correspondence:
Nicoletta Nicolaou:
n.nicolaou@imperial.ac.uk

1 SUPPLEMENTARY TABLES

Supplementary Table 1. Mean causality for Dataset 2. Results are from pairwise/univariate and conditional/multivariate estimates from linear GC, Kernel-GC and C_{NPMR}

GC pairwise		To: x_1	x_2	x_3	*GC* conditional		To: x_1	x_2	x_3
From:	x_1	—	0.688	0.279	From:	x_1	—	0.665	0.003
	x_2	0.002	—	0.502		x_2	0.003	—	0.226
	x_3	0.003	0.025	—		x_3	0.003	0.003	—

$K-GC$ univariate		To: x_1	x_2	x_3	$K-GC$ multivariate		To: x_1	x_2	x_3
From:	x_1	—	0.491	0.237	From:	x_1	—	0.476	0
	x_2	0.001	—	0.392		x_2	0	—	0.195
	x_3	0.001	0.018	—		x_3	0	0.001	—

C_{NPMR} pairwise		To: x_1	x_2	x_3	C_{NPMR} conditional		To: x_1	x_2	x_3
From:	x_1	—	0.614	0.172	From:	x_1	—	0.510	0 (-0.125)
	x_2	0 (-0.084)	—	0.422		x_2	0 (-0.003)	—	0.064
	x_3	0 (-0.018)	0.003	—		x_3	0 (-0.055)	0 (-0.018)	—
