# Supplementary material for: A Nonlinear Causality Estimator Based on Non-Parametric Multiplicative Regression
Source: Front Neuroinform. 2016 Jun 14;10:19. doi: 10.3389/fninf.2016.00019 (PMC4905976; doi:10.3389/fninf.2016.00019)
Supplement: Supplementary file 2 [file Table2.PDF]

Supplementary Material:
A nonlinear causality estimator based on Non-Parametric Multiplicative Regression

Nicoletta Nicolaou * and Timothy Constandinou

*Correspondence:
Nicoletta Nicolaou:
n.nicolaou@imperial.ac.uk

1 SUPPLEMENTARY TABLES AND FIGURES

Supplementary Table 1. Mean causality for Dataset 3. Results are from pairwise/univariate and conditional/multivariate estimates from linear GC, Kernel-GC and C_{NPMR}

GC pairwise		To: x_1	x_2	x_3	*GC* conditional		To: x_1	x_2	x_3
From:	x_1	—	0	0	From:	x_1	—	0.004	0.003
	x_2	0	—	0.078		x_2	0.003	—	0.085
	x_3	0	0	—		x_3	0.003	0.003	—
$K-GC$ univariate		To: x_1	x_2	x_3	$K-GC$ multivariate		To: x_1	x_2	x_3
From:	x_1	—	0.192	0.152	From:	x_1	—	0.158	0.131
	x_2	0.001	—	0.097		x_2	0.001	—	0.110
	x_3	0.002	0.004	—		x_3	0.001	0.001	—
C_{NPMR} pairwise		To: x_1	x_2	x_3	C_{NPMR} conditional		To: x_1	x_2	x_3
From:	x_1	—	0.067	0.047	From:	x_1	—	0.063	0.049
	x_2	0.001	—	0.083		x_2	0	—	0.085
	x_3	0.002	0.001	—		x_3	0.002	0	—
