# Supplementary material for: A Nonlinear Causality Estimator Based on Non-Parametric Multiplicative Regression
Source: Front Neuroinform. 2016 Jun 14;10:19. doi: 10.3389/fninf.2016.00019 (PMC4905976; doi:10.3389/fninf.2016.00019)
Supplement: Supplementary file 3 [file DataSheet1.PDF]

Supplementary Material: A nonlinear causality estimator based on Non-Parametric Multiplicative Regression

Nicoletta Nicolaou * and Timothy Constandinou
*Correspondence:
Nicoletta Nicolaou:
n.nicolaou@imperial.ac.uk

1 SUPPLEMENTARY DATA

Appendix 1 Non-Parametric Multiplicative Regression for prediction - numerical example.

The following is a numerical example of how NPMR can be modified for time-series prediction. Another example can also be found in Mccune (2011).

The example time-series contains the following $n = 10$ samples: $Y = \begin{bmatrix} 1 & 3 & 5 & 1 & 3 & 4 & 2 & 3 & 5 & 2 \end{bmatrix}$

Step 1. To assess the effect of the past of Y on the current value of Y, we need to create a matrix of predictors, $\mathbf{X}$, via time-delay embedding. For the particular example we set the embedding dimension $d = 2$; therefore, the time-delayed matrix of predictors, $\mathbf{X}$, is:

$$\mathbf{X} = \begin{bmatrix} 3 & 1 \\ 5 & 3 \\ 1 & 5 \\ 3 & 1 \\ 4 & 3 \\ 2 & 4 \\ 3 & 2 \\ 5 & 3 \end{bmatrix}, \text{ with the corresponding response variable, } Y = \begin{bmatrix} 5 \\ 1 \\ 3 \\ 4 \\ 2 \\ 3 \\ 5 \\ 2 \end{bmatrix}$$

We now have three new time-series, each with $T = n - d$ samples, i.e. $T = 8$. As we can see, $\mathbf{X}(t) = Y(t-1), T(t-2)$ are used as predictors of $Y(t)$.

Step 3. Set the tolerance of each predictor. Without losing generality we set $s_k = 1, (k = 1, ..., d)$ in this example.

Step 4. Recall equation 2:

$$\hat{y}_t = \frac{\sum_{i=1, i \neq t}^{T} y_i \left(\prod_{j=1}^{m} w_{ij} \right)}{\sum_{i=1, i \neq t}^{T} \left(\prod_{j=1}^{m} w_{ij} \right)}$$

where the weights w_{ij} are estimated with an appropriate weighting function. In this study we will use a Gaussian function (eq. 3): $w_{ij} = e^{-0.5\left[(x_{i,j} - x_{t,j})/\sigma_j\right]^2}$.

The first target point ($t = 1$) to be predicted is $Y(1) = 5$, and the corresponding predictor variables are $\mathbf{X}(1) = X(1,1), X(1,2) = 3, 1$. First, estimate the weights of each predictor at all time points, except $i \neq j$ (excluding $i = t$ corresponds to cross-validation and avoids overfitting):

$$w_{21} = e^{-0.5\left[(X_{2,1}-X_{1,1})/\sigma_1\right]^2} = e^{-0.5[(5-3)/1]^2} = 0.1353$$

$$w_{22} = e^{-0.5\left[(X_{2,2}-X_{1,2})/\sigma_1\right]^2} = e^{-0.5[(3-1)/1]^2} = 0.1353$$

Continuing for all remaining weight terms:

$$w_{81} = e^{-0.5\left[(X_{8,1}-X_{1,1})/\sigma_1\right]^2} = e^{-0.5[(5-3)/1]^2} = 0.1353$$

$$w_{82} = e^{-0.5\left[(X_{8,2}-X_{1,2})/\sigma_1\right]^2} = e^{-0.5[(3-1)/1]^2} = 0.1353$$

Then using equation 2:

$$\hat{Y}_1 = \frac{Y(2)w_{21}w_{22} + Y(3)w_{31}w_{32} + ... + Y(8)w_{81}w_{82}}{w_{21}w_{22} + w_{31}w_{32} + ... + w_{81}w_{82}}$$

$$= \frac{1 \times 0.1353 \times 0.1353 + 3 \times 0.1353 \times 0.0003 + ... + 2 \times 0.1353 \times 0.1353}{0.1353 \times 0.1353 + 0.1353 \times 0.0003 + ... + 0.1353 \times 0.1353}$$

Applying for all sample points, the response variable estimate $\hat{Y}$, i.e. the prediction of Y using X, is:

$$\hat{Y} = \begin{bmatrix} 4.199 \\ 2.199 \\ 3.004 \\ 4.776 \\ 2.542 \\ 3.147 \\ 3.659 \\ 1.622 \end{bmatrix}$$

Step 5. Estimate the variance of the absolute difference between Y and $\hat{Y}$.

The above is a description of how NPMR can be applied to predict a response variable from a given set of predictor variables that contain past information of the response variable itself and/or past information from a chosen set of predictor variables. To estimate the extent by which a response variable is affected by a particular response variable, the standard Granger causality estimation can be used and the autoregressive models are replaced by NPMR-based prediction (eqs. 5 and 6).
