# Supplementary material for: A Nonlinear Causality Estimator Based on Non-Parametric Multiplicative Regression
Source: Front Neuroinform. 2016 Jun 14;10:19. doi: 10.3389/fninf.2016.00019 (PMC4905976; doi:10.3389/fninf.2016.00019)
Supplement: Supplementary file 4 [file DataSheet2.PDF]

Supplementary Material: A nonlinear causality estimator based on Non-Parametric Multiplicative Regression

Nicoletta Nicolaou * and Timothy Constandinou

*Correspondence:
Nicoletta Nicolaou:
n.nicolaou@imperial.ac.uk

1 SUPPLEMENTARY DATA

Appendix 2. Non-Parametric Multiplicative Regression Sensitivity - numerical example.

The sensitivity, Q, is inherent in NPMR and can be used to evaluate how much each predictor contributes to the NPMR-based prediction. This is achieved by nudging each of the predictor values up and down and re-calculating the new response variable estimate. Higher values of Q indicate a bigger sensitivity of the response variable to a particular predictor.

The following numerical example has been adapted from McCune (2011). Recall the time-delay embedded time-series from Appendix S1:

$$\text{Predictors, } \mathbf{X} = \begin{bmatrix} 3 & 1 \\ 5 & 3 \\ 1 & 5 \\ 3 & 1 \\ 4 & 3 \\ 2 & 4 \\ 3 & 2 \\ 5 & 3 \end{bmatrix}, \text{ and response variable, } Y = \begin{bmatrix} 5 \\ 1 \\ 3 \\ 4 \\ 2 \\ 3 \\ 5 \\ 2 \end{bmatrix}$$

Step 1. Estimate the response variable without cross-validation, $\hat{y}$, i.e. using the procedure described in Appendix S1, but also including $i = t$:

$$\hat{y} = \begin{bmatrix} 4.492 \\ 1.760 \\ 3.001 \\ 4.492 \\ 2.350 \\ 3.053 \\ 4.133 \\ 1.760 \end{bmatrix}$$

Step 2. Each predictor is nudged by a small proportion, Δ, of the range of its values: $d_k = |x_{maxk} - x_{mink}|\Delta$; here we set $\Delta = 0.05$ without loss of generalisation. Hence, for the first predictor of $\mathbf{X}$: $d_1 = |5 - 1|0.05 = 0.2$. In this particular example it so happens that $d_2 = d_1 = 0.2$.

Step 3. Nudge the first predictor up and down by 0.2:

$$\mathbf{X}_{nudged} = \begin{bmatrix} 3+0.2 & 1 \\ 3-0.2 & 1 \\ 5+0.2 & 3 \\ 5-0.2 & 3 \\ . & . \\ . & . \\ . & . \\ 5+0.2 & 3 \\ 5-0.2 & 3 \end{bmatrix} = \begin{bmatrix} 3.2 & 1 \\ 2.8 & 1 \\ 5.2 & 3 \\ 4.8 & 3 \\ . & . \\ . & . \\ . & . \\ 5.2 & 3 \\ 4.8 & 3 \end{bmatrix}$$

Step 4. Estimate the response variable $\hat{Y}_{nudged}$ without cross-validation:

$$\hat{Y}_{nudged} = \begin{bmatrix} 4.452 \\ 4.514 \\ 1.713 \\ 1.848 \\ . \\ . \\ . \\ 1.713 \\ 1.848 \end{bmatrix}, \text{ where } \hat{y}^{+} = \begin{bmatrix} 4.452 \\ 1.713 \\ . \\ . \\ . \\ 1.713 \end{bmatrix} \text{ and } \hat{y}^{-} = \begin{bmatrix} 4.514 \\ 1.848 \\ . \\ . \\ . \\ 1.848 \end{bmatrix}$$

Step 5. Estimate the sensitivity, Q, using equation 7:

$$Q(Y, X_k) = \frac{\sum_{i=1}^{T} \left(|\hat{y}_i^{+} - \hat{y}_i| + |\hat{y}_i^{-} - \hat{y}_i|\right)}{2T|y_{max} - y_{min}|\Delta}$$

$$= \frac{(4.452 - 4.199) + (4.514 - 4.199) + ... + (1.716 - 1.760) + (1.848 - 1.760)}{2 \times 8|5 - 2|0.05}$$

For the given example: $Q_1 = 0.303$.

Step 6. Repeat steps 2-5 for the second predictor: $Q_2 = 0.255$.

In this particular example, the response variable is more sensitive to the first predictor.
