# Supplementary material for: A Nonlinear Causality Estimator Based on Non-Parametric Multiplicative Regression
Source: Front Neuroinform. 2016 Jun 14;10:19. doi: 10.3389/fninf.2016.00019 (PMC4905976; doi:10.3389/fninf.2016.00019)
Supplement: Supplementary file 5 [file DataSheet3.PDF]

Supplementary Material: A nonlinear causality estimator based on Non-Parametric Multiplicative Regression

Nicoletta Nicolaou * and Timothy Constandinou
*Correspondence:
Nicoletta Nicolaou:
n.nicolaou@imperial.ac.uk

1 SUPPLEMENTARY DATA

Appendix 3 Signal-dependent noise model.

Luo and colleagues consider the following first order AR-BEKK model for two univariate time series, x_t and y_t:

$$\begin{bmatrix} x_t \\ y_t \end{bmatrix} = \begin{bmatrix} 0.1 & 0 \\ 0 & 0.1\sqrt{2} \end{bmatrix} \begin{bmatrix} x_{t-1} \\ y_{t-1} \end{bmatrix} + \begin{bmatrix} r_{xy,t} \\ r_{yx,t} \end{bmatrix} ; \begin{bmatrix} r_{xy,t} \\ r_{yx,t} \end{bmatrix} = \begin{bmatrix} \Sigma^{1/2}_{xy,t/t-1} \epsilon_{xy,t} \\ \Sigma^{1/2}_{yx,t/t-1} \epsilon_{yx,t} \end{bmatrix} \tag{1}$$

where

$$\Sigma^{1/2}_{xy,t/t-1} = 1 + \begin{bmatrix} q_1 & (1-q_1)q_3 \end{bmatrix} \begin{bmatrix} x_{t-1} & y_{t-1} \end{bmatrix} \begin{bmatrix} x_{t-1} & y_{t-1} \end{bmatrix}^T \begin{bmatrix} q_1 & (1-q_1)q_3 \end{bmatrix}^T \tag{2}$$

$$\Sigma^{1/2}_{yx,t/t-1} = 1 + \begin{bmatrix} (1-q_2)q_4 & q_2 \end{bmatrix} \begin{bmatrix} x_{t-1} & y_{t-1} \end{bmatrix} \begin{bmatrix} x_{t-1} & y_{t-1} \end{bmatrix}^T \begin{bmatrix} (1-q_2)q_4 & q_2 \end{bmatrix}^T \tag{3}$$

and $\epsilon_{xy,t}$ is normally distributed noise; $q_i (i = 1, ..., 4)$ are uniformly distributed random numbers in the range $[0, 1]$. One can control the presence of a signal-dependent noise causal influence by setting $(1-q_1)q_3 = 0$ (causal influence $y_t \rightarrow x_t$), $(1-q_2)q_4 = 0$ (causal influence $x_t \rightarrow y_t$), or $(1-q_1)q_3 = (1-q_2)q_4 = 0$ (bidirectional causal influence $x_t \leftrightarrow y_t$).
