# Supplementary figures and images for: A Nonlinear Causality Estimator Based on Non-Parametric Multiplicative Regression
Source: Front Neuroinform. 2016 Jun 14;10:19. doi: 10.3389/fninf.2016.00019 (PMC4905976; doi:10.3389/fninf.2016.00019)

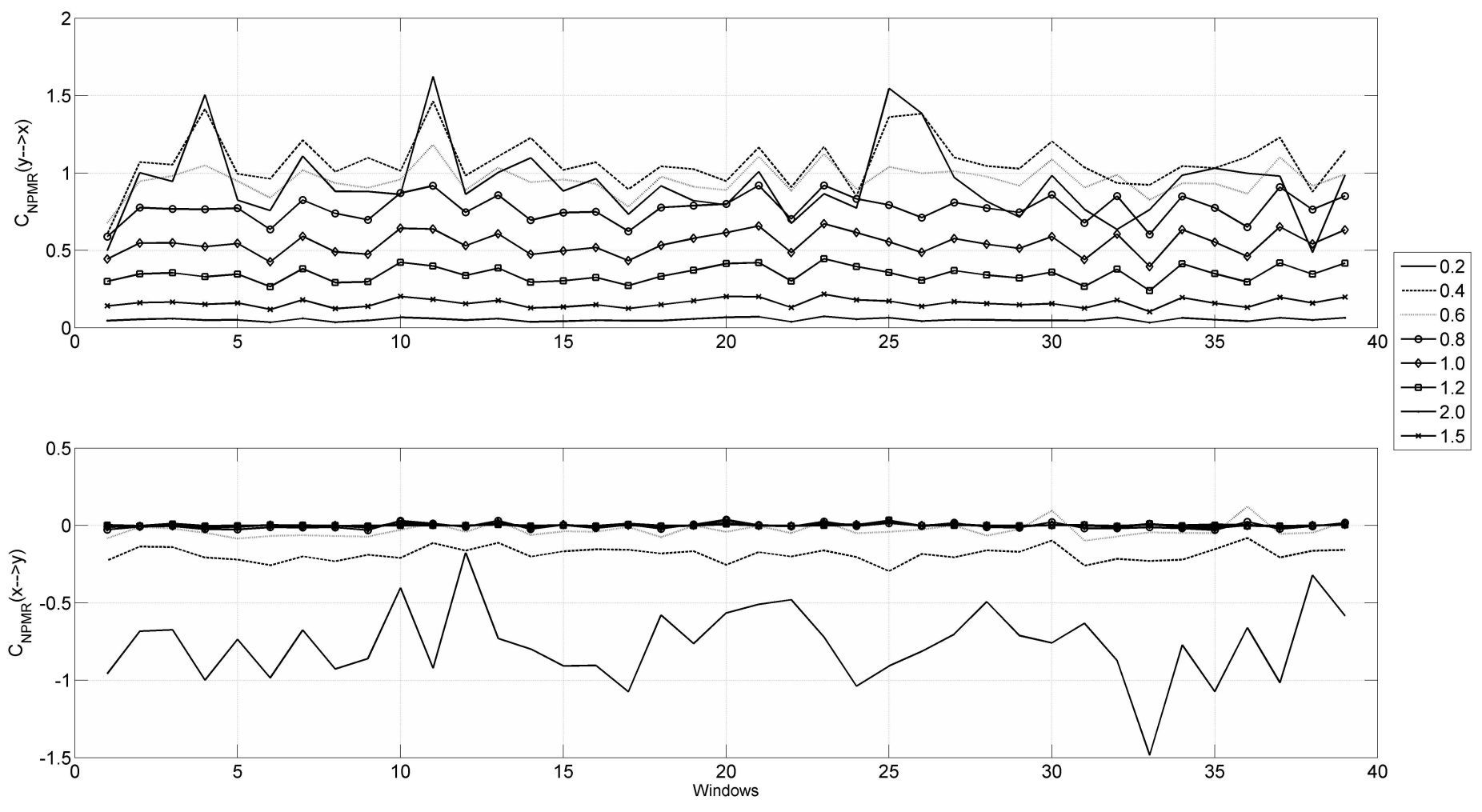

Supplement: Supplementary file 6 [file Image1.JPEG]

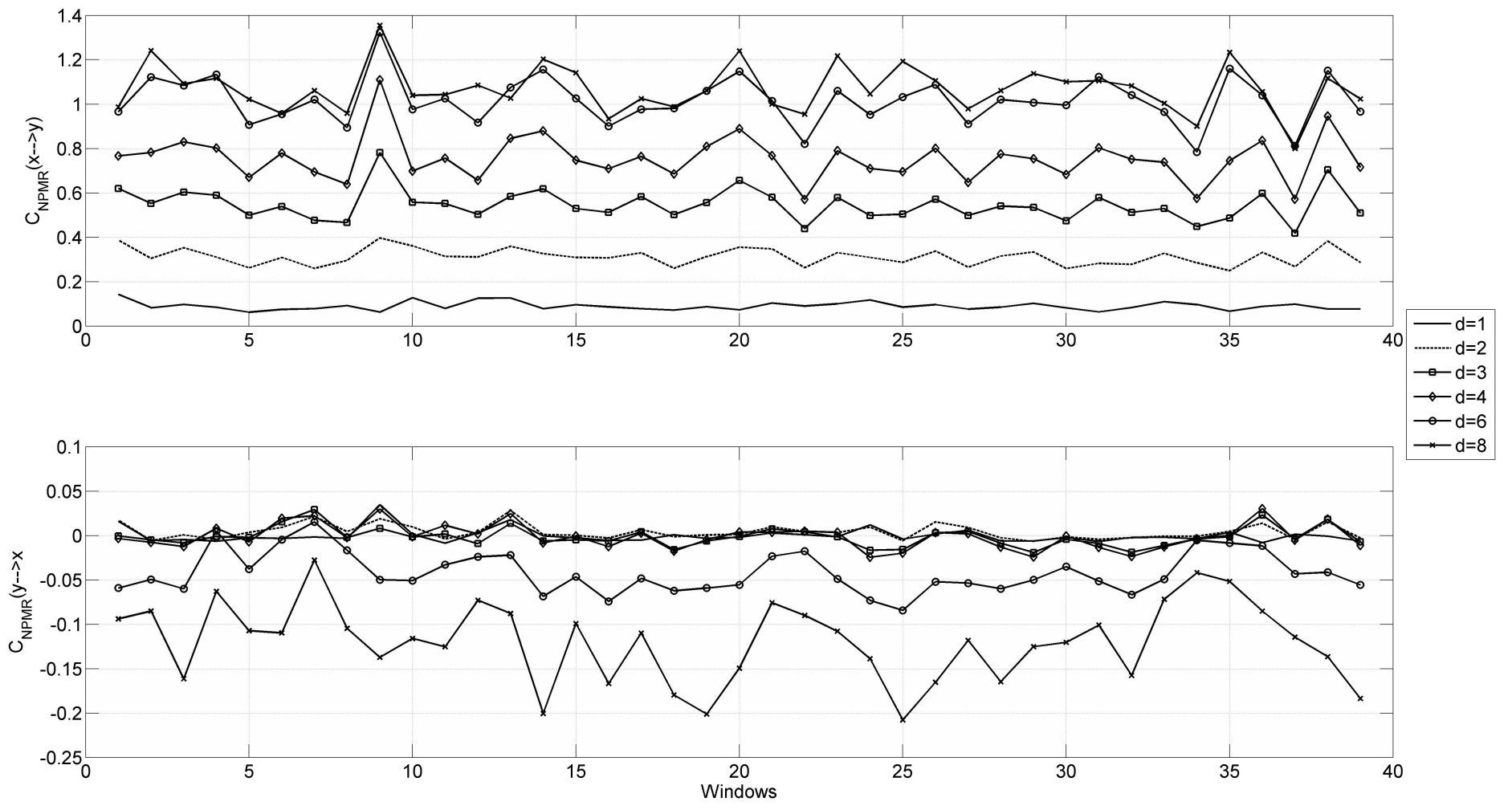

Supplement: Supplementary file 7 [file Image2.JPEG]
